# From Trophic Magnification Factors to Multimedia Activity Ratios: Chemometers as Versatile Tools to Study the Fate of Hydrophobic Organic Compounds in Aquatic Ecosystems

**DOI:** 10.1021/acs.est.4c07940

**Published:** 2024-11-11

**Authors:** Elisa Rojo-Nieto, Theo Wernicke, Melis Muz, Annika Jahnke

**Affiliations:** a Department of Exposure Science, Helmholtz Centre for Environmental Research - UFZ, Permoserstr. 15, Leipzig 04318, Germany; b Institute for Environmental Research, RWTH Aachen University, Aachen 52074, Germany

**Keywords:** passive equilibrium sampling, bioaccumulation, partitioning, thermodynamics, multimedia environment, hydrophobic organic compounds

## Abstract

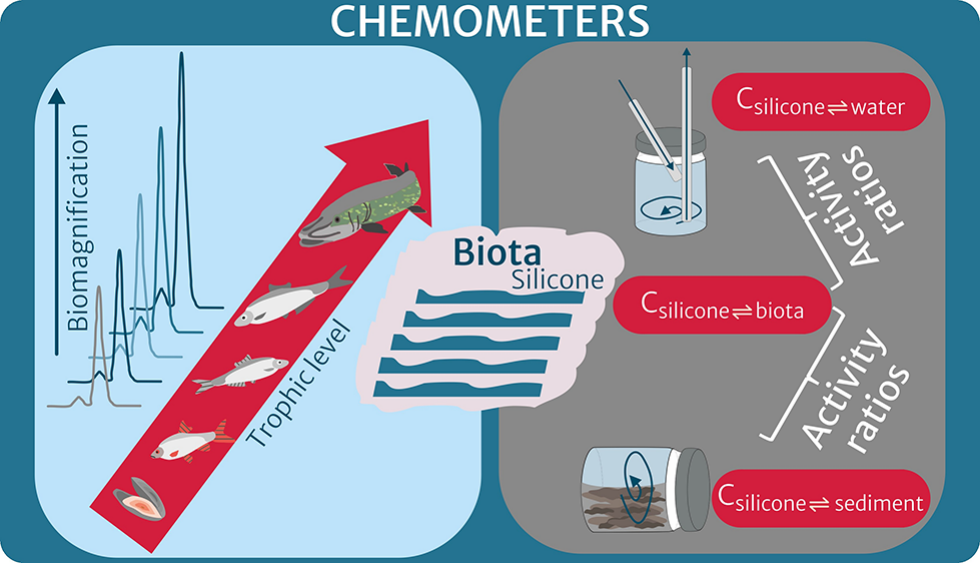

We applied passive equilibrium sampling using silicone-based chemometers to nine biota species, sediment, and water in a multimedia aquatic ecosystem. They allowed for direct comparison of the concentration of regulated and emerging hydrophobic organic compounds in the silicone across species as well as the comparison of biota with sediments and water. We derived chemometer-based trophic magnification factors (TMFs) of diverse compounds that agreed with the traditionally derived TMFs. Our exploratory work in water demonstrated that equilibrium with newly designed chemometers can be achieved in few days for compounds with a log *K*_OW_ up to 6. We calculated activity ratios, dividing the concentrations in the silicone equilibrated with biota by those equilibrated with the abiotic exposure media (sediments and water), assessing the thermodynamics of bioaccumulation and the equilibrium state between the ecosystem compartments. They confirmed that the biota were below equilibrium partitioning relative to sediments and water, as other studies have described. Silicone-based chemometers open up new opportunities and applicability in multimedia aquatic ecosystems for studies that rely on equilibrium partitioning of the *in-situ* mixtures of chemicals, such as multimedia assessments or application of effect-based methods.

## Abstract 2

This study applies silicone chemometers at equilibrium in a multicompartment aquatic ecosystem, calculating TMFs and activity ratios to assess thermodynamics between compartments.

## 1 Introduction

In chemical risk assessment, the (aquatic) bioaccumulation potential of substances is evaluated as a critical property. According to Kosfeld et al.,^1^ bioaccumulation assessment is nowadays mainly anchored in laboratory-based data (e.g., partition coefficients, the bioconcentration potential of substances in fish, the bioaccumulation of sediment-associated chemicals in endobenthic oligochaetes, and the bioaccumulation of chemicals in soil oligochaetes).^2−5^ These data have the benefit of being determined under controlled conditions and with limited cost, but they may lack environmental relevance. Therefore, the limited use of field data in regulatory bioaccumulation is unfortunate and needs more attention,^1^ given that complex environmental mixtures and other environmentally relevant conditions cannot be fully simulated in laboratory experiments.

The traditional way of determining bioaccumulation in field studies is to calculate the ratios of concentration of a substance in two or more media (e.g., biota and sediments) based on concentrations normalized to the major sorptive phase (for hydrophobic organic compounds, HOCs, these usually are storage lipids for biota and organic carbon for sediments and suspended particulate matter) to allow for comparisons. It is regularly described with standard parameters such as bioconcentration factors (BCF, uptake from the surrounding abiotic media), biomagnification factors (BMF, uptake via food), and bioaccumulation factors (BAF, uptake from all pathways). Those metrics can lead to some bias due to differences in extraction protocols and sorptive capacities of the main sorptive phases, which can also be, e.g., proteins in lean tissues.^6−8^ Aiming to circumvent that need for normalization and the associated bias, the chemometer approach^8^ is based on passive equilibrium sampling and direct comparison of the concentrations in the chemometer equilibrated with different media, without any further normalization. In this regard, equilibrium sampling devices have opened a new analytical window for determining chemical activity,^8,9^ which is a measure of the “effective concentration” of a species in a mixture and a driver for partitioning, biouptake, and toxicity. Working on a chemical activity basis allows us to express the data on a common basis while obtaining information on the freely dissolved fraction of the chemicals that is available to biota. Chemical activity is particularly useful to predict spontaneous processes to which the chemical may be subject, including bioaccumulation.

HOCs include a large group of pollutants, in some cases legacy and/or regulated and many contaminants of emerging concern (CEC) (e.g., polychlorinated biphenyls (PCBs), polycyclic aromatic hydrocarbons (PAHs), and polybrominated diphenyl ethers (PBDEs)).^10^ They are characterized by low water solubility, some of them being persistent and toxic, many being prone to bioaccumulation.^11,12^ Thanks to recent advances,^10,13^ it has become possible to use equilibrated chemometers for HOCs in all kinds of biota tissues, including lean tissues, making chemometers highly useful in bioaccumulation studies.^14^ To study bioaccumulation of pollutants in aquatic ecosystems, in particular across trophic levels, the trophic magnification factor (TMF) can be used.^15,16^ The TMF of a chemical describes its average accumulation in a food web. Hence, TMFs integrate bioaccumulation processes over an entire food web under realistic environmental conditions. It has been suggested in support of the risk assessment of marketed chemicals, which are already present in the environment and are subject to regulatory concerns.^1,15,17^ Despite the fact that they show some general patterns across different ecosystems, they are specific for each ecosystem. Thus, they need to be determined in each case for the accurate interpretation of local data. They have been considered as a tool for normalizing concentrations in different fish species for a common trophic level under the European Water Framework Directive (WFD),^1^ highlighting their relevance.

In this work, we focus on a small and shallow lake ecosystem with stable conditions (minor inputs of environmental chemicals, largely from the atmosphere, no stratification) and apply chemometers in biota from different trophic levels of the food web to directly determine the TMFs of HOCs based on the concentrations in the silicone chemometers. In TMF studies, a regression of biota concentration data against the species’ trophic positions, based on stable isotope ratios for nitrogen, is carried out. Usually, that regression uses the lipid-normalized pollutant concentrations, which we replace by the chemometer-based pollutant concentrations in the silicone following equilibration with the tissues of biota without the need of normalization, circumventing the associated bias. Furthermore, we advance the use of chemometers equilibrated with abiotic compartments (i.e., sediments and water) to allow for studying the multimedia environment and to calculate the activity ratios between biota and abiotic compartments. Regarding sediments and suspended particulate matter, there are well established methods for passive equilibrium sampling,^18−22^ but for water, it is still a challenge to approach equilibrium, especially for the more hydrophobic compounds.^23^ The activity ratios have the potential to characterize the concentrations of HOCs in biota with respect to their surrounding environmental media^14,24^ and allow to discern whether a specific trophic level is at equilibrium with an abiotic compartment or not, opening up the use of chemometers in abiotic compartments to assess aquatic bioaccumulation. In this study we (i) investigate the potential of chemometers (passive equilibrium samplers) to accurately determine TMFs of an aquatic ecosystem and (ii) use multimedia chemometers to determine the equilibrium status of biota toward their surrounding abiotic compartments using chemical activity ratios, with an established method for sediments and exploring a new approach aimed to reach equilibrium between silicone chemometers and water.

## 2 Materials and Methods

### 2.1 Study Area

The study site is Lake Ången (58°45′05″ N, 17°11′30′′ E), Sweden. This lake was selected due to previous data existing from this environment,^7,18^ being a well characterized and stable ecosystem. Details about the sampling campaign, carried out in September 2018, are provided in Text S1 and Figure S1 in the Supporting Information (SI).

### 2.2 Chemometers for Passive Equilibrium Sampling in Biota, Sediments, and Water

#### 2.2.1 In biota

Two types of tissues (muscle and whole body for fish, muscle for crayfish, and whole body for mussels) were studied covering nine different species (mussels (*U. tumudus* and *A. anatina*), crayfish (*P. leniusculus*), roach (*R. rutilus*), bream (*A. brama*), perch (*P. fluvialis*), pikeperch (*S. lucioperca*), pike (*E. lucius*), and eel (*A. anguilla*)). The set of samples (including two entire subsets: muscle and whole body) is detailed in Table S1 and includes 60 fish, 37 mussels, and 75 crayfish samples; some of them were pooled. For sampling with the silicone chemometers, the method described in Rojo-Nieto et al.^10^ was used. Briefly, silicone sheets (SSP-M823, 125, 250, and 350 μm thickness, Specialty Silicone Products, polydimethylsiloxane, PDMS) were precleaned with Soxhlet extraction using ethyl acetate (EtAc) for 20 h. One or three different thicknesses of silicone (with the same surface area) were used for each sample, depending on the amount and lipid fraction of tissues that were available. The maximum mass of silicone to be used in each tissue was determined according to the lipid content (and hence the total mass of lipids) to ensure negligible depletion (sampling less than 5% of the pollutants present in the sample),^9,25^ to avoid altering the equilibrium concentration and thus perturb chemical activity.

For the tissue sampling with chemometers using relocations,^10^ the samples were kept at 4 °C during the experiment for up to 7 days, and the relocation of the silicone sheets was carried out manually, every 2 h (6 relocations per day, static overnight). To ensure that equilibrium was achieved by this procedure in the whole set of samples, concentrations in three silicone sheets with different thicknesses were evaluated, among others, for the most challenging tissue, perch muscle, due to its low lipid content (0.6%, see Section 3.2 and Text S4).

After exposure, the silicone sheets were extracted twice with EtAc (1 mL of solvent per 0.1 g of silicone each) and spiked with the internal standards during the first extraction step. Then, a mild cleanup was carried out, using EMR cartridges (Agilent Technologies, USA) followed by a Primary-Secondary Amine (PSA) sorbent (Agilent Technologies, USA).^21,26^ Further details can be found in Section 3.2 and in SI Text S2.

#### 2.2.2 In Sediments

The sediments were collected by a professional diver, pooling the upper layer (5 cm) in collection jars. For this study, the method using silicone-coated jars *ex situ* (coated with Dowsil DC-2577 Low VOC, Dow Chemical Company, USA) as described in refs (7, 18, and 27) and in Text S3 was used. The jars had a volume of 125 mL and a coating surface of 88 cm^2^ (5.4 cm diameter and 5.2 cm height). The coating thicknesses were 1.0, 2.0, and 3.5 μm.

#### 2.2.3 In Water

Identical coated jars as those used for sediments were used for equilibration with water, but with different coating thicknesses of 0.6, 1.0, 2.0, and 3.5 μm (*n* = 2 each). To challenge the establishment of equilibrium partitioning, a novel device (figure S3) with three pumps was used to pump water at 0.08–0.12 L s^—1^ out of the jars on site with an estimated flow velocity of 4.4 cm s^–1^, while they were submerged in the water (94 h). The pumping aimed to reduce the water boundary layer (WBL) by creating turbulence and to speed up the uptake of HOCs in the silicone chemometer. This approach was first tested in a previous sampling campaign, indicating equilibrium for selected compounds of rather low hydrophobicity (α- and γ-hexachlorocyclohexanes).^28^ The extraction and analysis of the coated jars were performed as for the jars equilibrated with the sediments.

### 2.3 Chemical Analysis

All samples were analyzed by gas chromatography-high resolution mass spectrometry (GC-HRMS, QExactive, Thermo Fisher Scientific, Germany), as described elsewhere,^26,29−31^ and further details, including details about the reagents and preparation of analytical standards, are given in Text S2. The 75 target chemicals were grouped according to their properties and/or usage into eight categories for better visualization. These groups were (i) PCBs (*n* = 12), (ii) PBDEs (*n* = 4), (iii) pyrethroids (*n* = 5), (iv) synthetic polycyclic musks, nitro musks and musk-like fragrances (musks, *n* = 7), (v) organochlorine pesticides (OCPs, *n* = 11), (vi) PAHs (*n* = 22), (vii) other industrial compounds including antioxidants, industrial precursors, intermediates, and UV filters (others, *n* = 13), and (viii) compounds having branched or unbranched aliphatic chains (LongChain, *n* = 1), following the criteria established in Muz et al.^26^ Details of the analyzed target chemicals are given in Table S3. Method detection limits (MDLs) and QA/QC procedures are described in detail in Text S2 and Table S4 for biota and the method detection limits (MDL) for coated jars are available in the literature^29^ and in Table S8.

### 2.4 Calculation of the Trophic Level

For the determination of the trophic level (TL), the samples were homogenized, freeze-dried, and cryo-milled, and the nitrogen and carbon stable isotopes δ^15^N and δ^13^C were determined by an elemental analyzer interfaced with an isotope ratio mass spectrometer (EA-IRMS, Text S5 and Table S1). δ^15^N (ratio of the two stable isotopes of nitrogen,^15^ N:^14^N) in fish species is known to increase with their TLs and also to differ between ecosystems. This divergence is partly due to their dependence on the distinct value at the base of the food chain. Therefore, according to international guidelines, the δ^15^N for primary consumers, mussels, from the specific ecosystem of this study (called “baseline” in eq 1) has been used to calculate the site-specific TL of all the other species (secondary consumers)
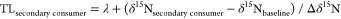
1with δ^15^N_baseline_ being the δ^15^N of the mussels from this ecosystem (two species, see Section 2.2), δ^15^N_secondary consumer_ the δ^15^N of the species under study, and the Δδ^15^N the shift in δ^15^N typical for one TL (3.4‰).^32^ λ is the trophic position of the baseline species, being 2 in this case.^32^

### 2.5 Calculation of Trophic Magnification Factors (TMFs)

TMFs represent an alternative to the traditional bioaccumulation metrics, an ecosystem-specific approach averaged over all covered species, hence referred to as the “gold standard”,^33,34^ that is increasingly used and integrates enrichment processes in an entire food web. According to Kosfeld et al.^1^ and Kidd et al.,^35^ certain criteria should be fulfilled (a) to calculate the TMFs in an appropriately selected food web in the ecosystem under study and (b) for assessing the validity of the TMFs. Those criteria have been applied in this study and include, among others, the following requisites: the use of a minimum TL range of 2.0 (e.g., 2.0 to 4.0) to analyze a fish whole-body, to use a reasonable balance of lower-TL versus higher-TL organisms, to ensure that the organisms are linked by diet through the food web, to determine the δ^15^N and δ^13^C stable isotopes, to detect the target compounds in all samples above the detection limit, and to ensure that all organisms are collected within an appropriate sampling period (e.g., one season). Further details can be found in Text S6.

In this study, the TMFs were calculated as follows: as the concentration of contaminants relative to the TL is an exponential function,^17^ for calculating the TMF the regression between TL (calculated using eq 1) and the logarithm of contaminant concentration is used (eqs 2 and 3)
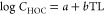
2
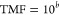
3with *C*_HOC_ being the concentration of the chemical in the tissue or the chemometer (ng kg^–1^) and “*b*” the slope of the linear regression when the concentration is expressed in logarithmic form.

### 2.6 Calculation of Activity Ratios between Environmental Compartments Using Chemometers

#### 2.6.1 Biota and Sediments

In the case of sediments and biota, the confirmation of equilibrium and the calculations of concentrations at equilibrium in the chemometers and in the major sorptive phases (lipids and organic carbon) are well established and published elsewhere.^7,10,13,14,19,20^

#### 2.6.2 Water

In the case of the water, it is still challenging to reach equilibrium since equilibrium was not approached even in prolonged sampling periods spanning weeks to months.^36^ Hence, usually, the concentration at equilibrium was extrapolated using performance reference compounds. We have used two approaches for calculating the equilibrium concentration in the chemometers exposed to water:

(a) To estimate if equilibrium was reached using our novel device, we evaluated if a linear regression between the mass of each compound and the mass of silicone in the different samplers with the same surface area but different thicknesses could confirm the attainment of equilibrium as is a regular procedure for sediments and biota (see details of this approach in Text S4 and Section 3.2). Until now, there are no samplers that allow to successfully apply this approach in water, not reaching equilibrium for most of the HOCs above an octanol/water partition coefficient, log *K*_OW_, of 5 even after months of exposure,^23^ but it has not been tested yet with the μm-thin silicone in the format of coated glass jars. In this approach, the slope indicates the concentration at equilibrium with the water media.
(b) We tested the calculation of the concentration at equilibrium using the contaminant mass ratio (CMR) calibration approach suggested by Fuchte et al.^37^ where two samplers with the same surface area but different thickness (i.e., different sampler mass) can be compared in the uptake phase (or at equilibrium), and from this comparison, the freely dissolved concentration in the medium at equilibrium (*C*_free_) can be estimated. For that approach, the chemometers cannot be in the very initial uptake phase, when the concentration is only dependent on the surface area and thus presenting similar mass of the compound in all the chemometers despite a different total mass of silicone. They need to be exposed to the media long enough to have absorbed different amounts of compounds as a result of different total mass of the samplers.

For the second approach, we applied the following equation and solved it numerically as well as graphically, using a numerical solution tool (Excel solver tool) and a graphic calculator (Geogebra.org):
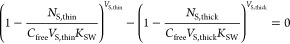
4where *N*_S,thin_ and *N*_S,thick_ are the accumulated mass in the thin and thick samplers (pg), respectively, *C*_free_ is the freely dissolved concentration in the surrounding medium (ng L^–1^), *V*_S,thin_ and *V*_S,thick_ are the volumes of the samplers (dm^3^), and *K*_SW_ is the sampler/water partition coefficient (L/L).^37^

### 2.7 Calculation of Activity Ratios between Environmental Compartments (Thermodynamics of Bioaccumulation)

To perform the comparison between environmental compartments accurately, we applied silicone:silicone partition coefficients (*K*_SilDC/SilSPP_)^38^ to compensate for the differences between the silicone polymers used for biota (SPP-M823) and for sediments (DC1-2577). Only those compounds present in the entire food web were evaluated in the sediments, and only those with an available *K*_*Sil*DC/SilSPP_ were translated to concentrations in SPP-M823, using eq 5
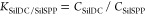
5

The concentrations of HOCs in the chemometers were used to calculate the ratios of chemical activities in biota (a_Biota_) relative to sediments (a_Sed_) or water (a_Water_) according to eq 6:
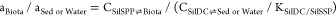
6where *C*_SilSPP⇌Biota_ is the concentration of the chemometers equilibrated with the biota, *C*_SilDC⇌Sed or Water_ the concentration of the chemometers equilibrated with the sediment or water, and *K*_SilDC/SilSSP_ the partition coefficient between the two polymers used as chemometers.

### 2.8 Modeling the Uptake of Chemometers in Water with Different WBL Thicknesses

To model the uptake of the studied chemicals into the μm-thin coatings of the chemometers under the scenario of different WBL thicknesses, we used the model proposed by Thompson et al.^16^ The model input parameters include *K*_SW_ (L L^–1^), diffusion in water, *D*_W_ (m^2^ s^–1^), and diffusion in the polymer, *D*_SIL_ (m^2^ s^–1^), for the compounds under evaluation. We have adapted the original script^16^ and applied it using Matlab Version 2024b (MathWorks, USA) as follows: as the jars are coated on their inner vertical walls (cylinder surface) and considering that the original model assumed no flux across the midline of the rectangular strip, so assuming two symmetric halves with a single exposure surface, the chemometers have been considered as one-half of a double-faced silicone strip. *K*_SW_ values have been taken from Gilbert et al.^38^ and Smedes,^39^ and *D*_W_ was calculated following the equation proposed by Schwarzenbach et al.^40^ (eq 7) as recommended in Lohmann^41^ and Booij.^42^*D*_SIL_ is not available in the literature for the DC1-2577 silicone that we applied, so for each compound, an average of *D*_SIL_ for Altesil (Altecweb, UK) and Silastic (Dow Corning, USA) silicones from Rusina et al.^43^ has been used (Table S9 and Text S7).
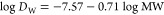
7where MW is the molecular weight (g mol^–1^) of the studied compound.

## 3 Results and Discussion

### 3.1 Food Web Structure of Lake Ången

Based on the stable isotope analyses of carbon (δ^13^C) and nitrogen (δ^15^N) in a full set of organisms from different trophic levels, the food web structure of Lake Ången was studied. The ratio of δ^15^N indicates the species’ trophic position, and δ^13^C indicates the dietary carbon source of biota in the freshwater reservoir. For the two species of mussels, crayfish, roach, perch, pikeperch, pike and bream (excepting three samples), the assumption of an increase of 0.4–1 ‰ δ^13^C per TL expected in food webs based on a single carbon source was valid,^44,45^ confirming the appropriate selection of the food web. Furthermore, in order to discern if the food web has been adequately characterized, we used the TMFs for PCB153 and DDE to confirm the appropriate characterization of the food web,^46^ as discussed in section 3.3. More details about the structure of the food web can be found in the SI, Figure S2.

### 3.2 Confirming Thermodynamic Equilibrium of Chemometers in Multimedia Environmental Compartments

#### 3.2.1 Biota

Out of the 75 HOCs studied, 20 were quantified in at least 90% of the samples (Table 3). Previous studies^10,13^ have demonstrated the achievement of equilibrium with the applied technique. Nevertheless, the achievement of equilibrium and the reproducibility of results was confirmed, using those samples with enough material to apply several chemometers in parallel with different thicknesses while ensuring negligible depletion.^20^ Concentrations in silicone were independent of the silicone thicknesses used, even in the most unfavorable cases of lean tissue (0.6% lipid content), one of the most challenging cases for equilibration, due to the lack of lipid droplets as transporter agents throughout the tissue.^10,13^ Further details can be found in Table S6 and Figure S4.

#### 3.2.2 Water

Using coated jars with different thicknesses for each sample to assess the equilibration status, we could confirm the attainment of equilibrium for pyrene (log *K*_OW_ 4.93), phenanthrene (log *K*_OW_ 4.35), and hexachlorobenzene (HCB) (log *K*_OW_ 5.86). Further details can be found in Table 1 and Figure S5. Furthermore, in the case of DDE and the PCBs studied, when checking the linear regression of the mass of silicone against the amount of analyte, it was observed that the equilibrium was not achieved for all four thicknesses but for the two thinner ones only (indicated in Table 1 as *“*different thicknesses: 2 (a&b)*”*). These two data points from the thinner chemometers allow drawing of the required regression line when forcing through the origin (0;0) (Table 1 and Figure S6). To compare these outcomes to the second approach, CMR, the concentrations in the silicone equilibrated with water were translated to concentrations in water at equilibrium, *C*_free_, using polymer–water partition coefficients available in the literature^38,39^ and in Table S9. In the second case, using the CMR calibration,^37^ the chemometers with different thicknesses were compared in pairs when the amounts of compounds sampled in each of them differed (see Section 2.6). Then, eq 4 was numerically and graphically solved. In Table 1, the results of this approach are given and compared with those of the linear regression approach. We observed a standard deviation (SD) of 1–14% of the average value of *C*_free_ in water obtained with both approaches, including also those cases where only the two thinnest silicone coatings were fully equilibrated with the media (those indicated with thicknesses “*a*” and “*b*” in the linear regression approach), except for PCB118, which presented an SD of 30%. Comparison of the two independent approaches hence showed that the results were in good agreement.

**Table 1:** C_free_ in pg L^–1^ Obtained Using the Contaminant Mass Ratio (CMR) Calibration Approach (Center) and the Linear Regression Approach (Right), Where “a” is the Nominal Thickness of 0.6 μm, “b” is 1 μm, “c” is 2 μm, and “d” is 3.5 μm. Italic Font Indicates Potential Underestimation of the Concentration Using the Pair “c-d” due to Concentration in “c”, See Figure S6

		Contaminant mass ratio (CMR) calibration approach						Linear regression approach	
		Different thicknesses paired						Diff. thicknesses (and (0,0))	
log *K*_OW_	Compound	a-d	a-b	a-c	b-c	c-d	**average**	2 (a&b)	4 (a,b,c&d)
5.86	HCB	31.1	31.1	31.1	29.0	27.7	**30.0**		28.9
6.98	PCB101	3.65	3.87	3.65	3.87	*2.20*	**3.45**	3.40	
6.34	PCB52	2.82	3.28	2.82		*1.67*	**2.65**	2.80	
6.00	DDE	5.09	5.46	5.09		*2.26*	**4.48**	4.80	
4.93	Pyrene	13925	13608	14637	14637	14637	**1428**		12700
4.35	Phenantrene	755	798	858	858	858	**825**		675
7.62	PCB138	0.60	0.56	0.56		*0.25*	**0.49**	0.48	
6.34	PCB44	1.43	1.43	1.43	1.36	*0.78*	**1.29**	1.24	
7.62	PCB153	1.45		1.45	0.84		**1.25**	1.18	
6.98	PCB118		0.90				**0.90**	1.39	

To further confirm that equilibrium was achieved for PCBs in the two thinner chemometers, we modeled the uptake in the scenario of different WBL thicknesses. For that purpose, we adjusted and applied the model proposed by Thompson et al.^16^ as indicated in Section 2.8 and in Text S7. Different thicknesses of the WBL (δ) have been modeled as follows: δ = 10 μm as a lower bound for turbulent systems, δ = 50 μm as an agitated laboratory system, and δ = 500 μm as an upper limit for WBL, considering a quiescent system.^16^ Three other intermediate scenarios have been considered, δ = 20 μm, δ = 30 μm, and δ = 100 μm. Even if some studies have calculated δ < 10 μm, we have kept the lower limit of the modeled WBL in δ = 10 μm for turbulent systems, in accordance with Lohmann^47^ and Thompson et al.^16^ among others. The graphical results for all the modeled δ can be found in Figure S10, and the estimated 95% equilibration time (*t*_95_) for the thinner WBLs, δ = 10 μm, δ = 20 μm, and δ = 30 μm, is included in Table 2.

**Table 2:** Calculated Times to Teach 95% of Equilibrium (*t*_95_) Using a Modified Version of the Model Proposed by Thompson et al.^16^

		t_95_ (d)	t_95_ (d)	t_95_ (d)
Compound	Sampler thickness (μm)	δ = 10 μm	δ = 20 μm	δ = 30 μm
PCB52	3.5	3.7	7.5	11.0
	2	2.1	4.2	6.3
	1	1.1	2.1	3.2
	0.6	0.6	1.3	1.9
PCB44	3.5	4.5	9.0	13.5
	2	2.6	5.2	5.2
	1	1.3	2.6	3.9
	0.6	0.8	1.6	2.3
PCB101	3.5	10.3	20.7	31.0
	2	5.9	11.8	17.7
	1	3.0	5.9	8.6
	0.6	1.8	3.5	5.3
PCB118	3.5	15.3	30.6	45.8
	2	8.7	17.5	26.2
	1	4.4	8.7	13.1
	0.6	2.6	5.2	7.9
PCB138	3.5	43.1	86.1	129
	2	24.6	49.2	73.8
	1	12.3	24.6	36.9
	0.6	7.4	14.8	22.1
PCB153	3.5	31.9	63.8	95.7
	2	18.2	36.5	54.7
	1	9.1	18.2	27.4
	0.6	5.5	10.9	16.4

The results of the modeling indicated that if we consider a turbulent system (δ = 10 μm) and an equilibration time of ∼4 days, the thinner chemometers will be close to reaching 95% of equilibrium for most of the compounds, except for PCB138, which in 4 days would be at 80% of equilibrium for the thinnest coating with δ = 10 μm. Similar near-to-equilibrium can be observed for PC52, PCB44, and PCB101 with δ = 20 and 30 μm. These results support the previous interpretation of the degree of equilibration in the different thicknesses of the silicone-coated jars and help to plan further modifications and improvements of this approach. This exploratory work opens up an approach for passive equilibrium sampling in water using silicone-coated jars. Equilibrium for compounds with log *K*_OW_ up to 6 and log *K*_SW_ up to 5.5 has been achieved with water in few days using silicone chemometers, extending the applicability of those devices.

#### 3.2.3 Sediments

For sediments, an *ex situ* passive equilibrium sampling approach has been well established for decades. Nonetheless, it is always recommendable to double-check that equilibrium has been achieved. In this study, the equilibration for each compound studied was confirmed by using jars with coatings of three different thicknesses for each sample. Further supplementary details can be found in Texts S3 and S4.

### 3.3 Trophic Magnification Factors (TMFs)

**Figure 1:**
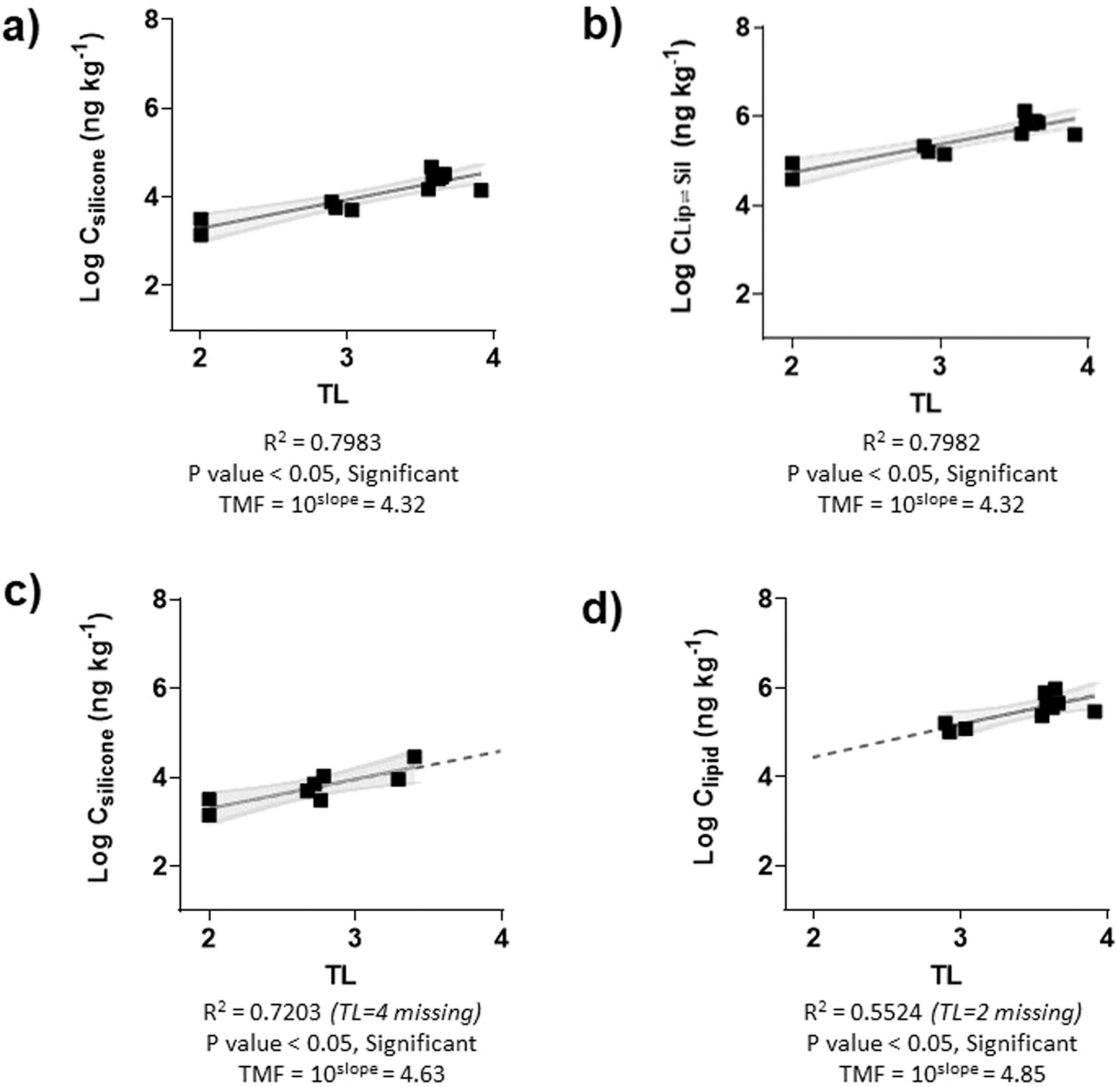
TMFs for PCB153 calculated using (a) the concentration in the chemometers equilibrated with muscle tissue homogenates, (b) the transformed chemometer concentration to lipid-based concentrations in muscle tissue homogenates, (c) the concentration in the chemometers equilibrated with whole body homogenates, and (d) the concentration obtained from exhaustive solvent extraction of muscle tissue (traditional approach). The gray areas represent the 95% confidence intervals. In (c) and (d), some trophic levels are missing, and the broken line indicates the extension of the calculated regression line, facilitating visual comparison.

Figure 1 shows the example of TMFs for PCB153, calculated (a) using directly the concentration in the chemometer equilibrated with muscle, *C*_silicone_ (Figure 1a), (b) transforming the concentration in the chemometer to lipid-based concentrations, *C*_Lip⇌Sil_, using the polymer–lipid partition coefficient from Jahnke et al.^48^ as follows: *C*_Lip⇌Sil_ = *C*_silicone_ ×*K*_Lip/Sil_ (Figure 1b), (c) using directly the concentration in the chemometer equilibrated with the whole-body, *C*_silicone_ (Figure 1c), and (d) using the concentration obtained through exhaustive solvent extraction in muscle tissue normalized by the samples’ lipid content, *C*_lipid_ (traditional approach, Figure 1d). It is important to note that (a) (muscle) and (c) (whole-body) were derived from different individuals from the ecosystem under study, not subsamples from the identical individuals, which adds further robustness to the findings. The four slopes that allowed the TMFs to be calculated were (a) 0.645, (b) 0.645, (c) 0.666, and (d) 0.686, and the corresponding TMFs were (a) 4.32, (b) 4.32, (c) 4.63, and (d) 4.85. The error propagation of the SD of the slope (σ_TMF_) for the four TMFs were (a) 0.45, (b) 0.45, (c) 0.78, and (d) 1.06. The comparability of the calculated TMFs using different approaches confirms that chemometers are useful tools to determine TMFs in aquatic ecosystems, allowing for direct comparison between trophic levels. TL = 4 or TL = 2 are missing in (c) or (d), respectively, because, in our sets of samples, those TL were not represented.

One of the prerequisites for the calculation of the TMF is to analyze the whole-body residues (see Section 2.5). In this study, we analyzed both whole-body and muscle samples. However, when the isotope analysis was carried out and the TL was determined for each sample, we found that the whole-body samples did not fulfill the minimum TL range spanning (a range of 2.0). To the contrary, the muscle samples spanned a wider range of TLs, fulfilling this essential requirement for the TMF calculations. Previous studies^49,50^ have shown that for some priority substances (including HOCs covered in the present study) the concentrations in muscle can be converted from fillet to whole fish by using conversion equations or factors, then being reliable to use the muscle (or fillet) for calculation of TMFs. Furthermore, this procedure is encouraged in some of the approaches of, for example, the EU WFD, where some of the environmental quality standards in biota (EQS_biota_) such as HCB, PBDEs, dioxins, and dioxin-like compounds should be assessed based on concentrations in the fillet.^51^ To assess the agreement of TMFs derived from muscle tissue vs whole-body, we compared both for the indicator compound PCB153, with the caveat that for the whole-body concentrations, the criterion of a minimum TL range of 2.0 was not fulfilled (Figure 1).

To fulfill the steady-state requirement^1,35^ (see Section 2.5), crayfish and eel were excluded from the calculations of the TMFs since the crayfish is an artificially introduced species and is kept in cages and the eel is migratory and has been artificially introduced in the ecosystem, and might not be from the same food chain (see Figures S2 and S9). According to Walters et al.^46^ specific indicator HOCs can be used to identify if the food web has been properly characterized, confirming one of the prerequisites for the study of TMFs in aquatic environments (see Section 2.5 and Text S6): it should be known that the organisms are linked by diet. Walters et al.^46^ state that the studies should include one or several benchmark chemicals that consistently exhibit biomagnification to ensure that all organisms derive the majority of their energy and the contaminant of interest from a relatively linear food chain. For this purpose, they suggest to use PCB153 (Figure 1) and/or 4,4′-DDE (Figure S7). PCB153 and 4,4′-DDE have relatively high TMF values that were always >1 according to their wide review,^46^ indicating that measured TMFs for other HOCs should be viewed with caution if PCB153 and/or 4,4′-DDE show an unusually low TMF in a particular food web.

Following the evaluation of TMFs for PCB153 in freshwater ecosystems from different studies (*n* = 28), Kosfeld et al.^1^ found the average value for PCB153 to be 3.2 ± 1.3 and for DDE 4.7 ± 1.9 (*n* = 34). In our study PCB153 showed a TMF of 4.4 and 4,4′-DDE of 6.6 being >1 and within the expected range, confirming that there is a relatively linear food chain fulfilling the criterion by Walters et al.^46^ This fact can also be inferred from Figure S2 and the δ^13^C increase per TL (see Section 3.1). Our data indicates the integrity of the study design and that the applied approach was fit for purpose.

So far, the available (aquatic) TMF data from different studies are mainly restricted to legacy substances, not covering other relevant compounds such as CECs.^1^ Applying chemometers and following this approach, TMFs for a wide variety of substances can be calculated, to extend the available TMF data, as for the 20 out of 75 substances studied in this work, which fulfilled all the criteria for the TMF calculation. Table 3 shows the 20 TMF values that we determined, according to the criteria described by Kosfeld et al.^1^ and Kidd et al.^35^ One of the most restrictive criteria in the case of our study was to have quantifiable levels of the compound in at least 90% of the samples, ideally in all of them. The TMFs in Table 3 ranged from 0.128 (pyrene) to 8.38 (PCB149) and were in a similar range of the TMFs described from other ecosystems^1,35,52−55^ excepting the case of PCB101, which was lower than expected (0.85, usually being above 1). To the best of our knowledge, this is the first study where the concentrations of the chemometers at equilibrium (*C*_silicone_) have been directly applied to determine the TMFs of a diverse set of regulated, legacy, and emerging compounds of a food web in a specific ecosystem, without applying further transformation or normalization of the data.

**Table 3:** TMFs in Muscle Tissue^a^

	log *K*_OW_	*n*	TMF	σ_TMF_	SE
2,2-Dimethoxy-2-phenylacetophenone	2.95	11	0.47	0.15	0.66
Diphenylmethane	4.01	12	0.44	0.05	0.23
4H-Cyclopenta[def]phenanthrene	4.60	11	0.29	0.11	0.59
1,1-dichloro-2,2-bis(4-methoxyphenyl) ethane	4.74	11	0.47	0.08	0.29
Prallethrin	4.88	12	0.45	0.07	0.35
Fluoranthene	4.93	12	0.13	0.02	0.37
Pyrene	4.93	12	0.16	0.03	0.37
Allethrin	5.52	12	0.44	0.03	0.14
*m*-Terphenyl	5.52	12	0.46	0.06	0.23
*p*-Terphenyl	5.52	12	0.49	0.06	0.21
4,4′-DDE	6.00	12	6.64	0.76	0.25
Tonalide	6.34	12	0.52	0.06	0.25
Cyhalothrin	6.85	11	0.52	0.11	0.43
PCB101	6.98	12	0.85	0.15	0.31
PCB118*	6.98	9	2.36	0.34	0.22
PCB138	7.62	11	3.49	0.41	0.20
PCB149	7.62	11	8.38	1.45	0.30
PCB153	7.62	12	4.42	0.45	0.22
PCB170	8.27	11	3.13	0.79	0.43
PCB180	8.27	11	2.81	0.50	0.31

a *n* is the number of samples used for the calculations (some of them representative of a pool of samples), SE is the standard error of the estimate (or SD of the residuals) and σ_TMF_ is the error propagation of the SD of the slope. log *K*_ow_ is the logarithm of the octanol-water partition coefficient (values were calculated using the U.S EPA’s EPI Suite (EPA, U. S.) v1.68). An asterisk indicates a compound below 90% detectability in the studied samples.

### 3.4 Activity Ratios between Biota and Abiotic Compartments

The application of activity ratios allows for a multicompartment assessment in multimedia aquatic environments and allows determining, among others, the thermodynamics of bioaccumulation and the equilibrium state between the compartments in the system. The activity ratios between biota and the two main abiotic exposure compartments, sediment and water, were explored. The equilibration of the chemometers with the different media was not carried out at the same temperatures, but were 4 °C for biota, 10–12 °C for water (*in situ*), and 15 °C for sediment porewater. In these cases, it is important to consider the influence of the temperature on the partition coefficients used. Jonker et al.^56^ quantified the effects of temperature on partitioning of HOCs to silicone rubber passive samplers and suggested equations for different compounds to derive it. The SD values of log *K*_SW_ in freshwater (0 ‰ salinity) due to different equilibration temperatures were calculated using the proposed equations, for the pairs 4–12 °C (biota–water) and 4–15 °C (biota–sediment) for the following compounds: fluoranthene, pyrene, PCB52, PCB118, PCB153, PCB138, PCB180, and HCB (table S10). The log *K*_SW_ SD (4 and 12 °C) ranged from 0.04 for HCB to 0.17 for PCB180 and from 0.06 (HCB and PCB52) to 0.24 (PCB180) in the case of log *K*_SW_ SD (4 and 15 °C). Those equations were defined for the silicone rubber Altesil, but according to the authors, similar behavior can be expected for other silicone rubbers. Given those SD, the influence of temperature on the partition coefficients shall be taken into account to interpret the following results by comparing different environmental compartments with caution.

#### 3.4.1 Biota vs Sediment

**Figure 2:**
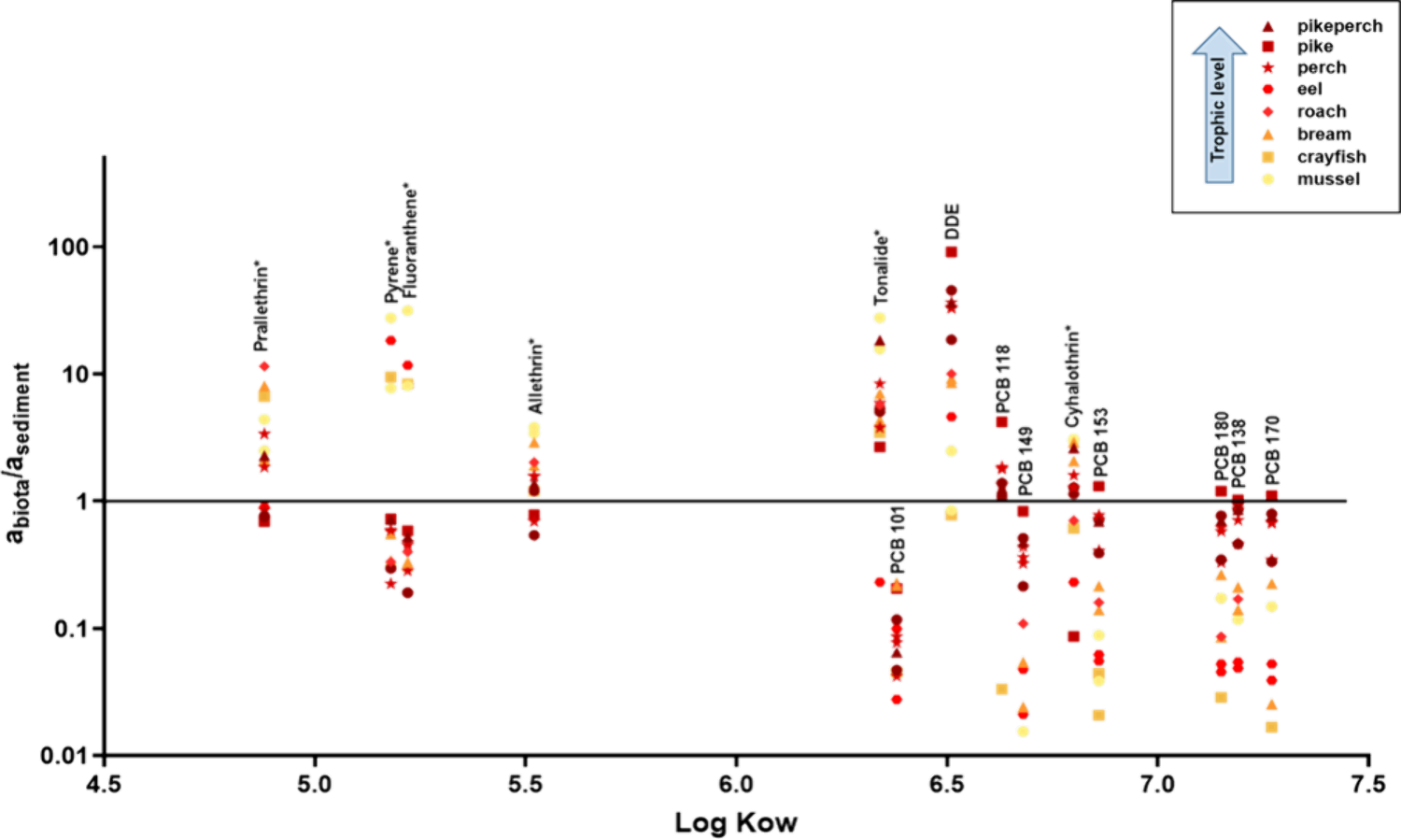
Activity ratios between biota and sediments. An asterisk indicates those HOCs for which an average *K*_SilDC/SilSPP_ was used.^18^

Activity ratios between biota and sediments were calculated by using eq 6 and are plotted in Figure 2. The concentrations in the silicone-coated jars equilibrated with sediments were averaged since no substantial differences were found between the different samples collected throughout the lake (SD ranging from 0.04 to 3.29 μg kg^–1^, being <35% of the average value for each compound). To compare both silicones, the partition coefficients *K*_SilDC/SilSPP_ were used, as indicated in eqs 5 and 6. Even if the *K*_SilDC/SilSPP_ from the literature, such as the ones from Gilbert et al.,^38^ can be used for exploratory work like in this study, their use is not ideal since they were obtained in a cosolvent setting, adding methanol to the water to foster equilibrium,^38^ which may affect the obtained partition coefficients, as discussed in Smedes.^39^ For extensive future studies, determination of *K*_SilDC/SilSPP_ without adding methanol is advised. For pyrene, fluoranthrene, pyrethroids, and tonalide, an average value from Wernicke et al.^21^ was used.

The compounds shown in Figure 2 have been selected due to their presence in all the samples of the different compartments and representing a wide variety of log *K*_OW_. For most of the PCBs, the biota/sediment activity ratios were <1 for most of the species, indicating a lower activity in biota relative to sediment, and only in the case of some high trophic level species, it exceeded 1. This indicates that the biota and the sediment were not at equilibrium until a TL around 3.5–4.0. This phenomenon has been observed earlier in this and other aquatic ecosystems, where only species of high trophic levels approached a ratio of 1 or above for highly hydrophobic HOCs.^7,14,21,57^ Some of the studied compounds clearly showed biomagnification along the food web. That contradicts the assumption of the basis of the food web being equilibrated with the surrounding media (and thus having an activity ratio of ∼1) and biomagnification across the food web bringing the upper trophic levels well above 1. Smedes et al.^14^ observed that as the hydrophobicity of HOCs increased, the biota was underequilibrated relative to the abiotic compartments, and at TL = 1, the concentrations in biota could be substantially lower than the thermodynamic equilibrium with the water phase, with deviations by up three orders of magnitude for compounds with very high *K*_OW_. This was also the case for the PCBs and other compounds in our study. The sediments under study were collected from the upper 5 cm layer. Given that the rate of sedimentation in lakes is on the order of millimeters per year, those samples can be considered an average of any seasonality that could exist in the sediments of Lake Ången. Furthermore, even if some seasonality might exist, the samples of the different media (water, sediment, and biota) were taken within 4 days, so the intersample comparison would be equally affected. The chemometers equilibrated with the concentration in the porewater measured the available concentration in the sediments under study (in contrast to the extractable concentration of the sediments obtained from exhaustive extractions). Smedes et al. hypothesized that in primary producers, HOCs are taken up from the aqueous phase by diffusion through an aqueous boundary layer at the surface of a cell wall or a membrane, and for HOCs of high hydrophobicity, mass transport in the water boundary layer controls the uptake rate. They modeled the system and found that the time required for equilibration exceeded the life span of the algae for compounds with an elevated hydrophobicity. This observation could explain why, consistently across different environments, like in this study, we did not find concentrations for certain HOCs at the level of thermodynamic equilibrium with the surrounding abiotic environment for biota below TL = 3 or 4 although biomagnification clearly occurred.

In the case of the PAHs of this study, the activity ratios were higher in invertebrates, without a clear correlation with the trophic level for the rest of the TLs. This pattern can be explained by the different capacities for metabolizing PAHs of fish and invertebrates, which may lead to lower bioaccumulation of those HOCs in fish tissues opposed to invertebrates.^11^ However, the activity ratios were higher than expected. Despite the fact that some studies describe BAFs in the order of one hundred for those compounds included in our study, such high values are not regularly observed.^58−60^ The same holds true for biota-to-sediment accumulation factors (BSAFs).^61−64^ One hypothesis could be that given the shallowness of the lake, photodegradation of the PAHs might occur in the abiotic compartments or other transformations in sediments, but further studies should be done regarding chemometers and PAHs in this ecosystem to confirm those activity ratios and the potential reasons behind. In the case of DDE, the activity ratios in the different biota studied were higher for the higher trophic levels, similar to the trends in PCBs, but with most of the biota species showing a ratio above 1. Besides accumulation from the environment and through the trophic web, the presence of DDE in biota can also be the result of biotransformation of accumulated DDT. For Tonalide (musk), Prallethrin, and Allethrin (pyrethroids), there is no or an inverse relationship of the activity ratios and the trophic level, in consonance with the TMFs found, with the activity ratio in most of the cases being above 1, too. During the sampling, we were cautious not to use any insect repellent, and we wore gloves at all times during sample handling. Furthermore, the field blanks did not reflect that the sampling campaign itself could have been the source of any of those compounds in the studied set of samples. However, the influence of the regular activities of the users of the lake cannot be ruled out as the potential source of some of these compounds in the study area, with no known sources of pollutants other than atmospheric input.

#### 3.4.2 Biota vs Water

**Figure 3:**
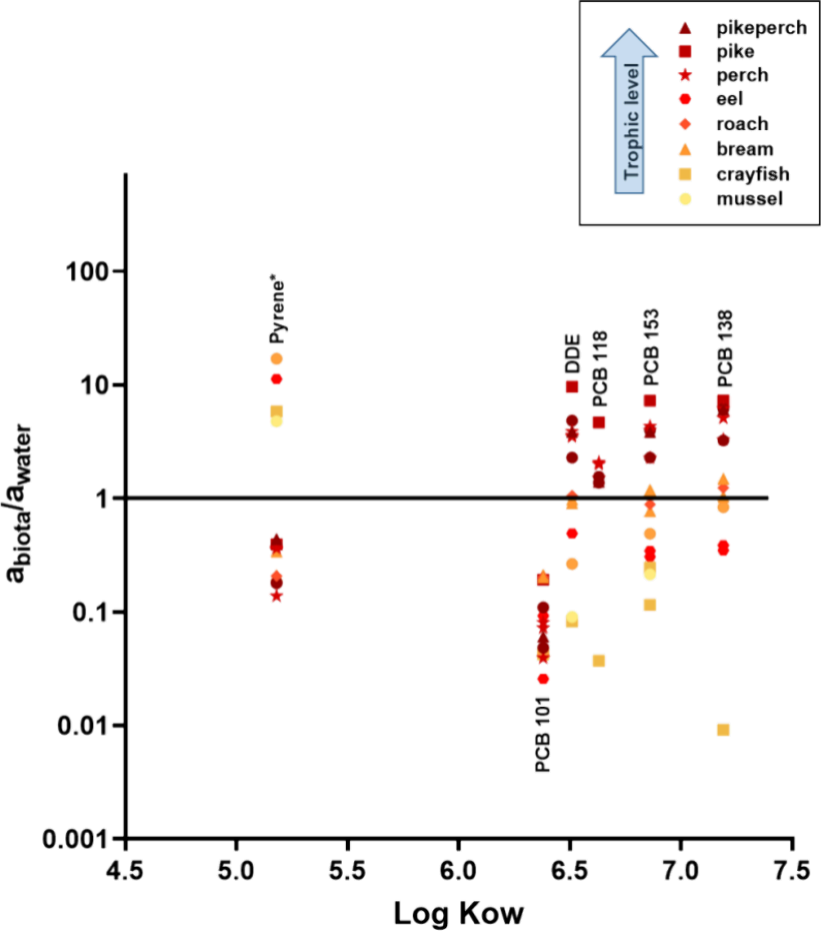
Activity ratios between biota and water. An asterisk indicates those HOCs for which an average *K*_*Sil*DC/SilSPP_ was used.^18^

As in the case of the sediments, activity ratios were calculated according to eq 6 following translation to the same silicone as the one used for biota, as described in eq 5; they are shown in Figure 3. The concentrations determined in the chemometers equilibrated with water were used for the calculation of the activity ratios related to biota. In the case of the activity ratios for this pilot study in the water compartment, they were calculated for the following 6 model compounds: pyrene, 4,4′-DDE, PCB101, PCB118, PCB153 and PCB138.

Similar to the sediments, for the PCBs in medium to high trophic level species, the activity ratio was above 1, whereas biota of lower trophic levels were underequilibrated with respect to the water. Despite following the same patterns as in sediments (Figure 2), there was a shift toward an activity ratio of ∼1 or above in the case of water for some of the PCBs. This shift has also been observed previously in another work from a different ecosystem.^21^ This first trial of this approach needs further study; therefore, any absolute results (such as the activity ratios) should be interpreted with caution.

For the five compounds in this study present in all the compartments studied (pyrene, PCB101, PCB 18, PCB153 and PCB138) the data indicate a higher chemical activity in sediment than in water with increasing log *K*_OW_ when those two compartments are compared. For them, the chemical activity ratios can be directly calculated since the same polymer was used. However, the reduced number of compounds and the exploratory approach in the case of water are not robust enough to formulate a solid conclusion in this regard. In addition, given the time span of the water sampling (only few days), some influence of seasonality cannot be ruled out. Despite the uncertainties of an approach still needing further development, these results in water and the potential of using the concentration in those chemometers to investigate the activity ratios between biota and water in addition to biota and sediment, as well as direct comparisons between water and sediment, are of relevance for aquatic monitoring and risk assessment. The approach presented in this study opens up the possibility of achieving equilibrium *in situ* using passive sampling in water, helping to evaluate the thermodynamics of HOCs within and between compartments in complex aquatic ecosystems.

## 4 Implications and Next Steps

The results from this study may have further implications for monitoring and risk assessment that should be thoroughly explored, such as the potential use of activity ratios in multicompartment evaluations. The results corroborate previous evidence that although biomagnification clearly occurred, the concentrations of certain HOCs reached equilibrium with the surrounding abiotic environment only at TL= 3 or 4. Jahnke et al.^18^ suggested that risks to wildlife and human health associated with bioaccumulation of chemicals from sediment could be more effectively assessed and managed site-specifically on the basis of chemical concentrations in model lipids at thermodynamic equilibrium with sediments (C_Lip⇌Sed_) as a conservative proxy of biomagnification. The results of this study support the utility of chemometers and related information that can be directly obtained, such as C_Lip⇌Sed_ or C_Lip⇌Water_, as a measure of the thermodynamic potential of abiotic compartments for the bioaccumulation of HOCs that could be used to make management decisions about contaminated sediments and their potential remediation. Further steps shall focus on using the same polymer for passive equilibrium sampling across media, avoiding the use of partition coefficients to translate concentrations from one polymer to another and the errors this might imply, as well as facilitating the applicability of this approach. To allow direct comparison of concentrations in silicone at equilibrium with different media and calculation of activity ratios, using the same polymer for passive sampling in the different environmental compartments or application of more robust polymer-to-polymer partition coefficients is a must.

The water passive equilibrium sampling device tested in this study achieved equilibrium for HOCs up to log *K*_ow_ 6 within few days, pending further improvements of the setup this approach may open up new possibilities to achieve *in situ* passive equilibrium sampling in water. That is especially interesting for studies involving bioassays, for example, which rely on equilibrium partitioning for realistic mixtures of compounds with diverse physicochemical properties. More work is needed in this direction, e.g., to increase the detectability of the chemicals under study by changing the geometry of the samplers and increasing the fraction of compounds that reach equilibrium partitioning within a reasonable time frame. In future work, it may be helpful, for further analysis and interpretation of the results, to include performance reference compounds during sampling. This exploratory work represents the first steps toward increasing the sensitivity and broadening the range of compounds that can be fully equilibrated using this approach.

## Supporting Information Available

Additional sampling and experimental details, including texts and diagrams of the sampling campaign, experimental setup, food web structure, TMF determinations and modeling of the uptake in water passive samplers (Supporting Information_1) (PDF)
Supporting tables containing the details of the target chemicals and their concentrations, dimensions of the chemometers, method detection limits, partition coefficients, and parameters used for the modeling (Supporting Information_2) (XLSX)
