# Supplementary material for: From Trophic Magnification Factors to Multimedia Activity Ratios: Chemometers as Versatile Tools to Study the Fate of Hydrophobic Organic Compounds in Aquatic Ecosystems
Source: Environ Sci Technol. 2024 Nov 11;58(47):21046–57. doi: 10.1021/acs.est.4c07940 (PMC11603764; doi:10.1021/acs.est.4c07940)
Supplement: Supplementary file 1 — es4c07940_si_001.pdf [file es4c07940_si_001.pdf]

From trophic magnification factors to multimedia activity ratios: chemometers as versatile tools to study the fate of hydrophobic organic compounds in aquatic ecosystems

Elisa Rojo-Nieto[a] *, Theo Wernicke[a], Melis Muz[a] and Annika Jahnke[a, b]

[a] Department of Exposure Science, Helmholtz Centre for Environmental Research - UFZ, Permoserstr. 15, 04318 Leipzig, Germany

[b] Institute for Environmental Research, RWTH Aachen University, 52074 Aachen, Germany

*Email: elisa.rojo-nieto@ufz.de

Summary: 21 pages, 7 Texts, 10 figures.

SUPPORTING INFORMATION

TABLE OF CONTENTS:

Text S1. Sampling campaign	Page S2
Figure S1: Map of Lake Ången, Sweden	Page S2
Figure S2: Food web structure of Lake Ången according to $\delta^{13}C$ and $\delta^{15}N$ stable isotopes	Page S3
Figure S3: Water sampling device	Page S3
Text S2: Reagents, materials and GC-HRMS analysis	Page S4
Text S3: Chemometers for passive equilibrium sampling in sediments	Page S5
Text S4. Confirming equilibrium	Page S5
Text S5: Stable Isotope Analysis	Page S6
Text S6: Criteria for calculating Trophic Magnification Factors (TMFs)	Page S7
Figure S4: Chemometers equilibrated with biota	Page S9
Figure S5: Chemometers of different thicknesses equilibrated with wate	Page S9
Figure S6: PCBs in chemometers of different thicknesses aiming for equilibrium with water	Page S10
Figure S7: Selected examples of the calculation of the TMFs using the concentration in the chemometers. The grey area represents the 95% confidence bands.	Page S10
Figure S8: Calculation of the TMFs using the concentration in the chemometers	Page S11
Figure S9. Graphical representation of the linear regressions which allow the calculation of the TMF for PCB153, with and without eel and crayfish.	Page S12
Text S7: Modeled uptake in the chemometer for water sampling with different thicknesses of the water boundary layer.	Page S12
Figure S10: Mass uptake into the silicone chemometer for water sampling with different thicknesses of the water boundary layer	Page S13

Text S1. Sampling campaign

The study site was Lake Ången (58°75'15'' N, 17°18'31'' E), a small (2.4 km^2 area) and shallow (5 m average and 8.5 m maximum depth, which ensures vertical mixing) Swedish lake southwest of Stockholm, with no known sources of HOCs other than the atmosphere. The lake is connected to the Baltic Sea through a narrow 200 m long stream. This lake has been selected due to previous data existing from this environment, being a well characterized and stable ecosystem. The sampling campaign was carried out in September 2018, deploying a) a passive equilibrium device for studying HOCs in water, b) sampling sediments (1-5 cm layer) manually, by a professional diver, at 6 different locations across the lake and c) collecting biota. The biota samples were obtained by fishing using a multimesh net and by manual collection, in case of the invertebrates. Mussels were collected by the diver and crayfish were collected from cages immersed in the lake, which is a regular activity carried out by the owners of the lake. Eels were sampled using eel traps by the owners and colleagues from the Swedish University of Agricultural Sciences and Stockholm University.

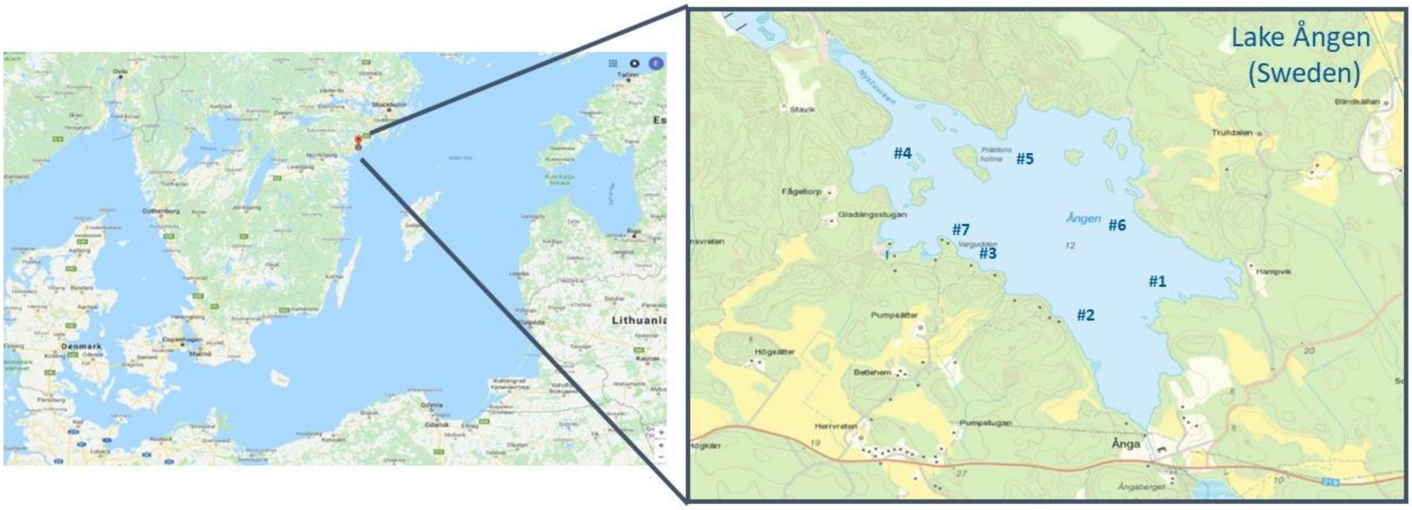

Figure S1. Map of Lake Ången, Sweden. Sediment sampling was carried out at stations #1 to #6. Fish nets were deployed in different areas of the lake, as well as the eel traps and crayfish cages. Water equilibrium device was deployed at location #7.

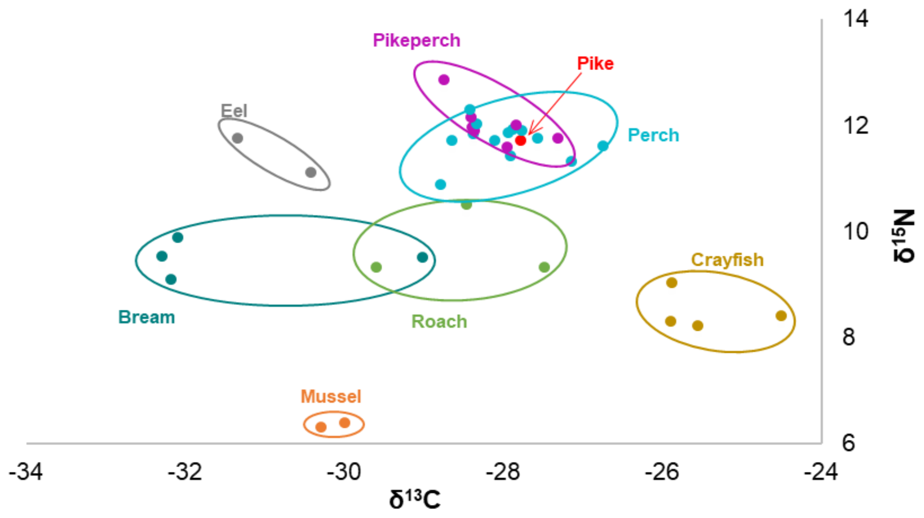

Figure S2. Food web structure of Lake Ången according to $\delta^{13}C$ and $\delta^{15}N$ stable isotopes.

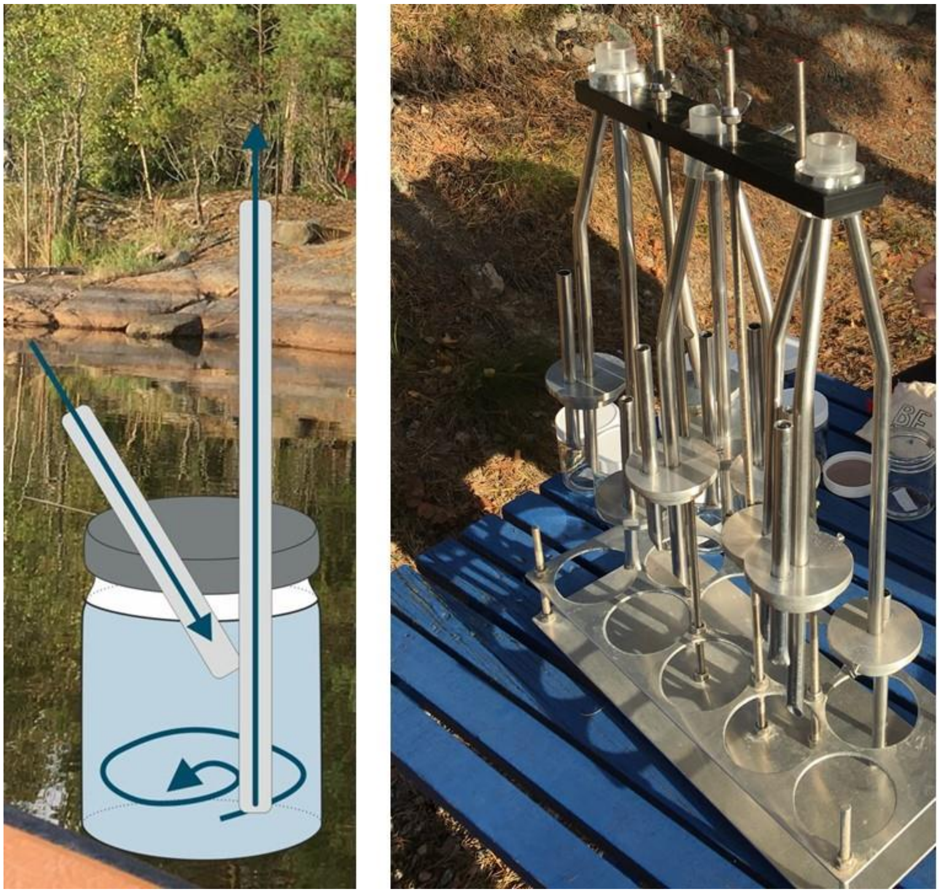

Figure S3. Water sampling device, using silicone-coated jars.

Text S2. Reagents and materials and GC-HRMS analysis.

The standards were purchased from Sigma Aldrich (Darmstadt, Germany) and Dr. Ehrenstorfer (Augsburg, Germany) with a purity of >98%. GC grade acetonitrile and GC-MS grade EtAc, 2-propanol, diethylether, and *n*-hexane were purchased from Merck (Darmstadt, Germany). The Captiva EMR-Lipid Cartridges (3cc) were purchased from Agilent (Folsom, CA, USA). The standard solution to prepare the calibration of the 75 environmental contaminants was prepared at 0.5 ng/µL in EtAc. The target chemicals were grouped according to their properties and/or usage into eight categories for better visualization. These groups were: (i) PCBs (*n*=12), (ii) PBDEs (*n*=4), (iii) pyrethroids (*n*= 5), (iv) synthetic polycyclic musks, nitro musks and musk-like fragrances (musks) (*n*=7), (v) organochlorine pesticides (OCPs, *n*=11), (vi) PAHs (*n*=22), (vii) other industrial compounds including antioxidants, industrial precursors, intermediates and UV filters (Others) (*n*=13) and (viii) compounds having branched or unbranched aliphatic chains (LongChain) (*n*=1), following the criteria established in Muz et al.[1] Details of the analyzed target chemicals are given in Table S3. Method detection limits (MDLs, given in Tabled S4 and S8) were calculated using fish oil as a matrix + 1 g silicone eq. extract, spiked with standard solutions at 0.1, 1, 10 and 100 ng/mL (*n*=4 at each level). The MDLs of the optimal method were estimated using a two-tailed t-distribution test with 99% confidence interval, based on the US EPA guidelines (EPA 2011). QA/QC procedures were in place for that instrument and the analysis method, including standard operating procedures, trained technicians dedicated to the equipment, traceability (including analytical standards and reference materials whenever available), etc.

To quantify the chemical concentration in the extracts, a gas chromatography-high resolution Orbitrap mass spectrometry (GC-HRMS) measurement was performed. A 13-point calibration, ranging from 0.1 to 1000 ng mL^{-1}, as well as solvent blanks and silicone blanks were prepared with the same method as the samples. Every batch that was run with the GC-HRMS contained the calibration and solvent blanks (prepared with the same cleanup procedure as the measured samples), 3-6 procedural silicone blanks and 12-18 samples. Blank subtraction was carried out in all the batches and all the samples (average+2SD). In addition, the 100 ng mL^{-1} -calibration point was injected in the middle of the batch and served as quality control, for the same reason the calibration concentrations 0.1, 1, 10, 100 and 1000 ng mL-1 were additionally measured at the end of every batch. Separation of the 117 target chemicals was achieved with a GC system consisting of a TriPlus RSH autosampler with a Trace 1310 GC coupled with a Thermal Desorption Unit (TDU-2) and a Cooled Injection System (CIS, both from Gerstel, Mülheim, Germany). The injections were made in splitless mode with an injection volume of 2 µL. Helium was used as carrier gas at a constant flow of 1.2 mL/min. The thermal desorption in the TDU was carried out with the heating program from 30 °C to 300 °C at a heating rate of 300 °C/min (holding 5 min). The transfer temperature of the TDU on the top of the CIS was set at 320 °C. After refocusing in the glass liner with deactivated glass wool (CIS-4 TDU, Gerstel, Mülheim, Germany) at -25 °C for 0.2 min, the analytes were desorbed with a temperature of up to 300 °C at a fast rate of 12 °C/s and a final holding time of 10 min and injected in a splitless mode with a time of 2 min. The chromatographic separation was based on the following temperature program: 60 °C (1 min), up to 150 °C at 30 °C /min, up to 186 °C at 6 °C/min rate, up to 300 °C with a rate of 4 °C/min (holding 11.5 min). The GC was coupled with a QExactive instrument (Thermo Fisher Scientific, Germany) via a transfer line kept at 280 °C. The ion source temperature was set at 250 °C. Mass spectrometric analysis was performed using electron ionization (EI) at 70 eV in positive

polarity, in full-scan mode with a scan range of 70-810 m/z and a resolution of 60,000 (FWHM at m/z 200). The internal calibration and tuning of the instrument were established using Perfluorotributylamine (PFTBA) as a mass calibrant. The data was processed and peak areas were integrated using the software Tracefinder General Quan 5.1 (Thermo Fisher Scientific). The quantification of the target compounds was based on an internal standard calibration method. 21 isotope labeled compounds were used, selected according to their retention time and chemical family to calculate area ratios to the target compounds (Table S3).

QA/QC chemical analysis: There is a long-term record and evaluation of tuning and calibration of the instrument (using 103 perfluorotributylamine (PFTBA) for mass calibration and tuning of the instrument), and method detection limits (MDLs) were determined. In this specific case, QA/QC of the samples was carried out running replicates (it is recommended that at least 10% of the samples are analyzed in duplicates) and a quality control sample (repeated standard injections after a predefined number of samples with a nominal concentration of 50 ppb) was run repeatedly with every batch, to check for cross contamination and a possible decline in instrument performance, allowing also to evaluate any shift in the retention times. In addition, selected calibration points (0.1, 1, 10, 100 and 1000 ng mL^{-1}) were measured at the end of the sequence of every batch. The sequences included procedural blanks, and instrument blanks were run at the beginning, following every three sample injections and in the end of the sequence. The samples were spiked with a mixture including all the target compounds at a given concentration. The positive identification of the compounds was based on the presence of the isotope "quantifier" m/z and two additional fragments m/z to confirm the compound ("qualifiers") at a given retention time.

Text S3. Chemometers for passive equilibrium sampling in sediments

For this study, 125 mL glass jars (Duran Wheaton Kimble, Germany) were internally coated with Silicone (Dowsil DC-2577 Low VOC, Dow Chemical Company, USA). Different amounts of Silicone were applied, *i.e.*, 8.22 ± 0.58, 16.62 ± 0.60, and 33.45 ± 2.2 mg (average ± standard deviation gravimetrically determined after cleaning), to obtain coatings of approximately 1.0, 2.0, or 3.5 µm thickness, respectively (average ± standard deviation 0.84 ± 0.06, 1.70 ± 0.06 and 3.47 ± 0,25). For equilibrium sampling 80 g of wet sediment was added to a solvent-cleaned and air-dried coated glass jar. Blank jars were prepared by adding 10 g of deionized water. To each jar, approximately 0.1 g of sodium azide was added to stabilize the sample and preclude any biological activity during further processing. The jars were rotated in the darkness at 8pm during 3 weeks. Then, the extraction was carried out with 2 mL EtAc (at least 1 mL of solvent per 0.1 g of silicone), spiked with the internal standards (IS), repeated once with an additional 2 mL aliquot of EtAc (no IS added) and the combined extracts were concentrated using an XcelVap (Biotage, USA) for chemical analysis.

Text S4. Confirming equilibrium

The achievement of equilibrium and the reproducibility of results was confirmed, ensuring negligible depletion. This approach consists of equilibrating silicone sheets of different thicknesses but the same surface area and evaluating the linear regression of the concentration of the compound in the chemometers against the mass of the silicone, forcing the linear regression through the origin (coordinates (0;0)).[2] This approach is based on the principle that several chemometers with different volumes (*i.e.*, different mass) but identical effective surface areas present different masses of the

chemical under study during the different uptake phases (assuming the uptake follows first order kinetics). At equilibrium, the amount of compound is proportional to the volume of the chemometer, which allows the confirmation of achievement of equilibrium evaluating the linear regression of the mass of the compound in the chemometer relative to the mass or the volume of the chemometer [2].

In their work, Reichenberg et al. [2] explain that equilibrium sampling with multiple polymer coating thicknesses (the one applied in the current study) can also reveal several sampling artefacts such as polymer abrasion and hence losing of silicone mass, surface adsorption, and sample depletion. The proportionality between the amount of analyte and the mass (or volume) of silicone for the different thicknesses used confirms valid sampling also for those cases. When loss of polymer exists during sampling (mainly due to abrasion), the amount of analyte would be less than the one fitting with the linear regression passing through (0;0). In this study it has been also double checked weighting the coated jars before and after the sampling, once clean and dry, confirming that there were not changes in the silicone mass due to abrasion. If surface adsorption happens, it would contribute to an extra amount of the mass of the analyte independently of the polymer volume, showing higher masses to volume than the ones fitting with the expected linear regression. In the case of potential depletion, it would lead to a reduction of the mass of analyte when comparing with the mass or volume of the polymer, showing a curve towards higher polymer masses. Reichenberg et al. [2] argue that when the amount of analyte measured in several samplers with same surface and different mass can be connected by a straight line passing through the origin, this is a strong indication of a successful and valid equilibrium considering all the previous exposed potential situations.

Text S5. Stable Isotope Analysis

To prepare samples for stable isotope analysis, homogenized tissue samples were freeze-dried, cryomilled and weighed into tin capsules (3.5 mm × 5 mm, HEKAtech, Germany) (~ 1 mg). Samples were analyzed in triplicates. A EuroEA3000 elemental analyzer (HEKAtech, Germany) was used for the combustion of the samples to N_2 and CO_2 gas. Flash combustion was performed with a 10 mL O_2 pulse in a commercially available combustion reactor filled with wolfram oxide, and silver cobalt oxide (HE46820995, HEKAtech, Germany) kept at 1050°C. The formed combustion product gases were swept with the helium carrier gas (gas flow = 80 mL/min) through a reduction furnace filled with copper kept at 650 °C. The remaining water was trapped with phosphorus pentoxide. Afterward, the gases were separated isothermally at 70 °C on a GC column (HE26070500, HEKAtech, Germany) and transferred into the IRMS via a ConFloIV open split system. Isotope ratios of an element (E) were expressed as delta notation ($\delta^{13}C$ or $\delta^{15}N$) in parts per thousand (‰) relative to the international standards (Eq. 1). The international standards were VPDB (Vienna Pee Dee Belemnite) for carbon and air for nitrogen, respectively.

$$\delta \mathrm{E}_{sample} = \left(\frac{R_{sample}}{R_{standard}} - 1\right) \times 1000 \tag{1}$$

R_{sample} and $R_{standard}$ are the isotope ratio ($^{13}C/^{12}C$ or $^{15}N/^{14}N$) of the sample or the corresponding ratio of the standard, respectively. Normalization of measured isotope compositions to international isotope-delta scales was done by analyzing reference materials in the same way as the samples and then applying a two-point calibration approach. The international reference materials used for normalization were: IAEA-CH6 (-10.45‰), IAEA-CH7 (-32.15‰) and IAEA-CH3 (-24.72‰) for carbon

and AS-3 (-0.35‰) and AS-11 (+11.0‰) for nitrogen. The analytical precision (SD of multiple runs) was below ±0.2‰ for both $\delta^{13}C$ and $\delta^{15}N$.

Text S6: Criteria for calculating the Trophic Magnification Factors (TMFs)

According to Kosfeld et al.,[3] for calculating the TMFs in an appropriate food web in the ecosystem under study, the following aspects should be considered:

(a) The selected food web items should be a representative, central part of the entire food web (*i.e.*, the sampled species should have a high abundance and express a dietary connection). The selection of the species has been based on previous work in the studied area.[4,5]
(b) Ideally, TMFs are derived along a distinct food web with one common source of nutrients at the base of the food web, in order to ensure a maximum dietary connection of the biota samples taken from the ecosystem. This aspect will be further evaluated in our selected food web, according to Waters et al.,[6] through the use of indicator compounds (see. Results and Discussion, sections 3.1 and 3.2).
(c) Before the sampling campaign is conducted, information about the species' diversity, abundance, and feeding habits in the ecosystem should be available. As for the first aspect, the selection of the species has been based in previous works in the studied area.[4,5]
(d) To fulfill the steady-state requirement, no migratory species should be included in the sample list as their chemical concentrations and stable isotope ratios may reflect habitats beyond the observed ecosystem. In our study, we correspondingly excluded crayfish and eel during the data evaluation for the TMF calculations, since the crayfish is kept in cages and the eel has been artificially introduced in the ecosystem, and might not be from the same food chain. Their data are included in the raw data in the supplementary information. They were included for the activity ratios with other abiotic compartments, but not used for the calculation of the TMFs.
(e) The selected species should not be endangered to obtain the necessary sampling permits. In the case of our study, the eels were artificially introduced in the ecosystem, and the permits for fishing them were obtained.

Together with those, according to Kidd et al.,[7] the following criteria should ideally also be fulfilled for assessing the validity of the TMFs generated using the novel chemometers:

(a) use a minimum trophic level (TL) range of 2.0 (*i.e.*, TL 2.0 -4.0). In this study our range of TL goes from 2 to 3.9.
(b) analyze fish whole-body residues. This study analyzes both, muscle samples and whole-body samples.
(c) apply an appropriate normalization (*e.g.*, to lipid or dry weight). This has been applied when needed (Details in section 3.3).
(d) use a reasonable balance of lower-TL versus higher-TL organisms. That criterion is fulfilled in this work (Details in table S2)
(e) the organisms should be known to be linked by diet through the food web. That link has been confirmed through the use of $\delta^{13}C$ and HOC indicators, as described in Walters et al.[6] (Details in section 3.3).
(f) $\delta^{15}N$ and $\delta^{13}C$ stable isotope data available. In this study, the stable isotopes were analyzed in all the samples (Table S1).

(g) appropriate baseline organism used for TL determination. The selection of the mussels as baseline organisms was done according to Deutsch et al.,[8] the Guidance Document No. 32 on biota monitoring (the implementation of Environmental Quality Standards for biota (EQS_{biota})) under the WFD.

(h) target compounds in all samples above the detection limit and adequate analytical quality data reported. In this study the TMFs have been calculated only for those HOCs present in all or at least 90% of the samples studied.

(i) all organisms collected within an appropriate sampling period (*e.g.*, one season). For this work, the organisms under study were collected in a time frame of 4 d.

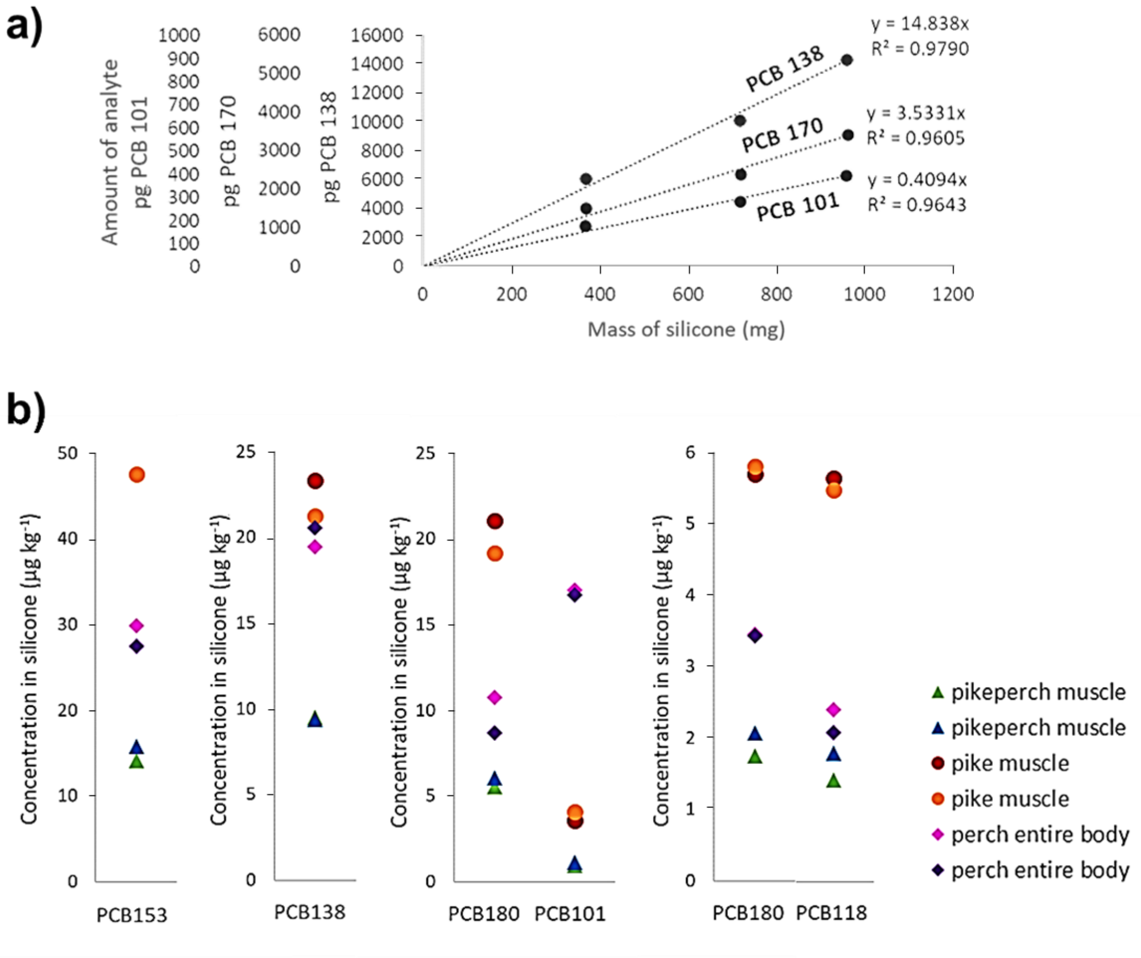

Figure S4. Chemometers equilibrated with biota. a) Chemometer data for three model compounds in different thicknesses of silicone equilibrated with perch muscle (0.6% lipid content) and the associated linear regressions, confirming equilibrium. b) Duplicates of chemometers equilibrated with tissues of a variety of lipid content.

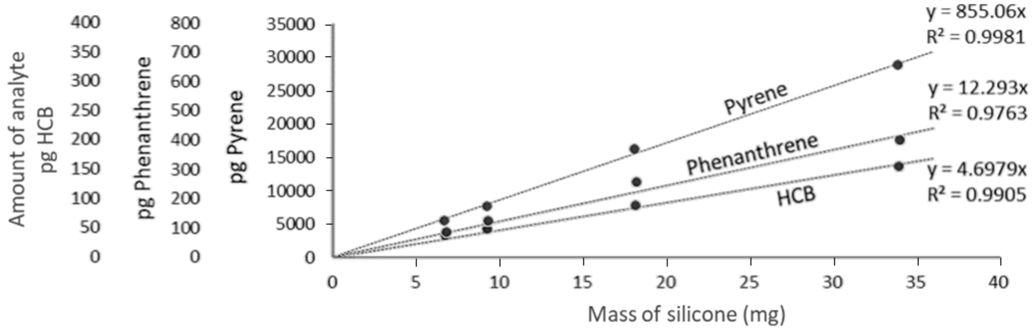

Figure S5. Linear regression of the amount of analyte respect versus to the silicone mass in chemometers of different thicknesses equilibrated with water.

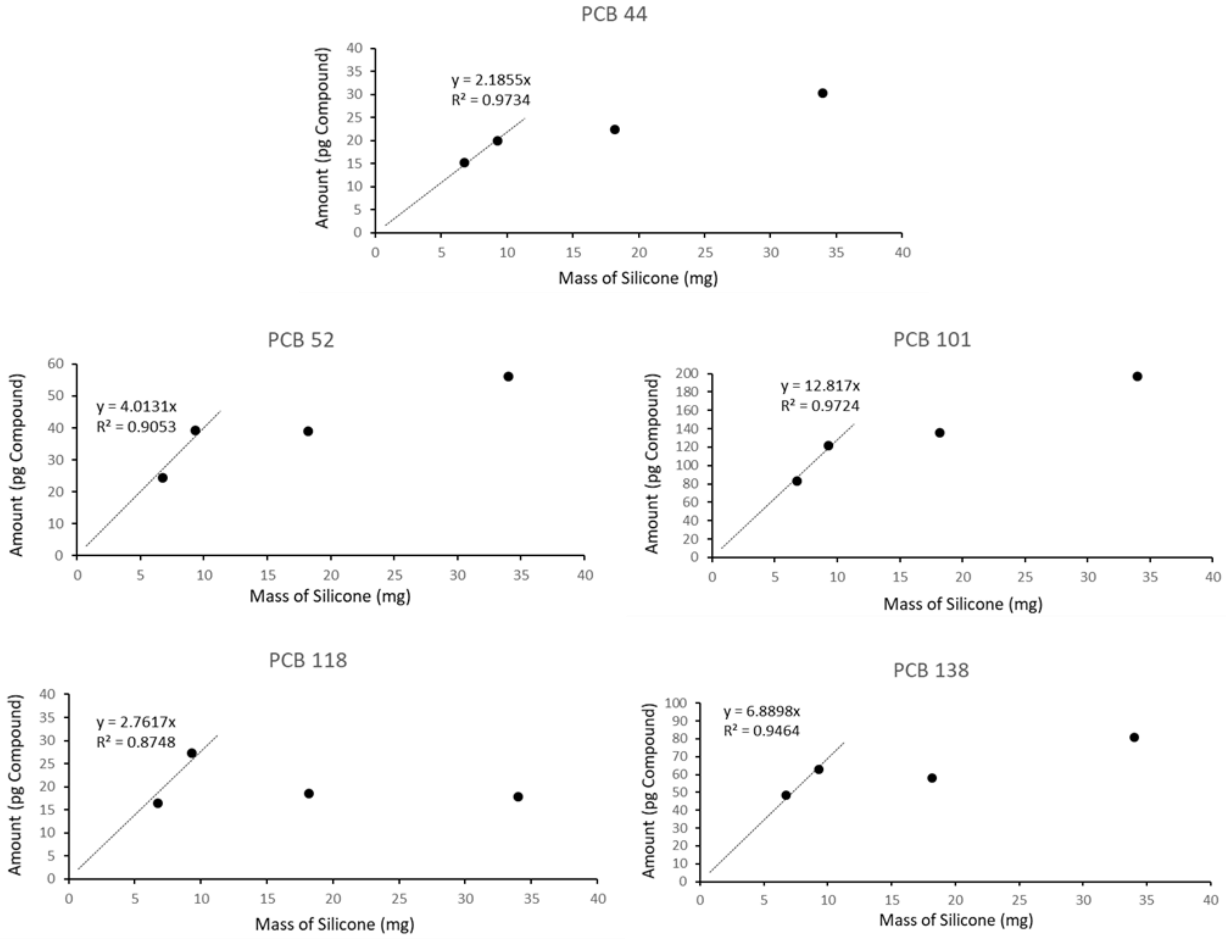

Figure S6. Linear regression of the amount of PCBs respect to the silicone mass in chemometers of different thicknesses aiming for equilibrium partitioning with water.

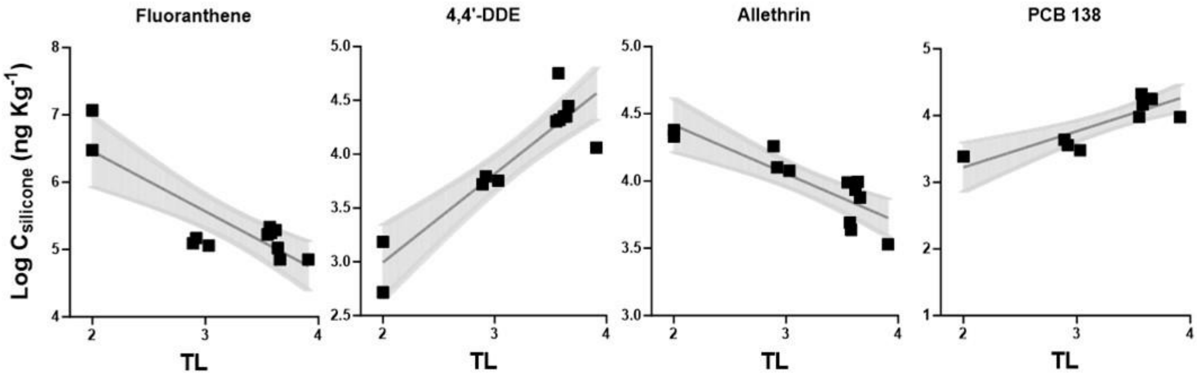

Figure S7. Selected examples of graphical representation of the linear regressions which allow to calculate the TMFs, using the concentration in the chemometers. The grey area represents the 95% confidence intervals.

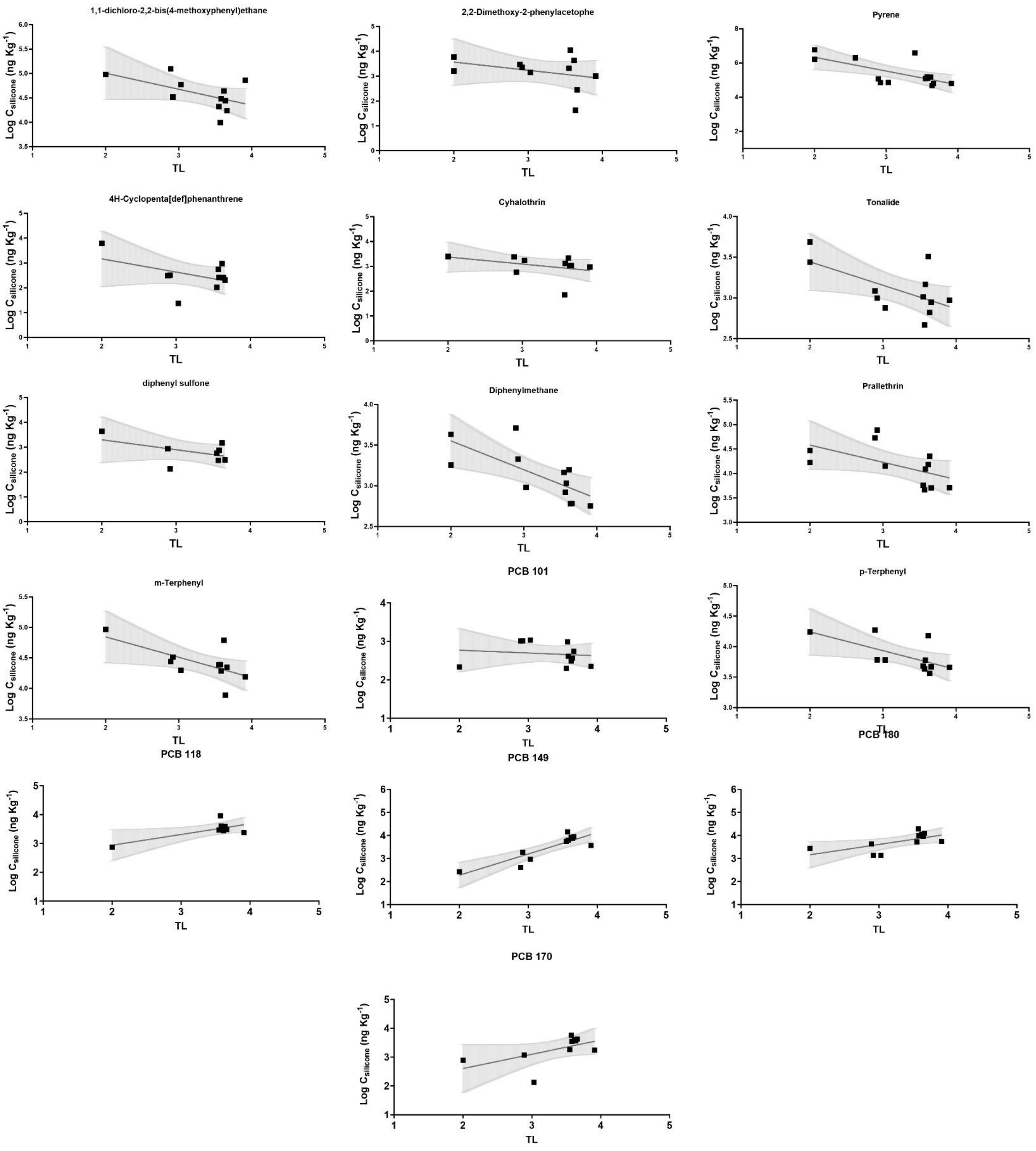

Figure S8. Graphical representation of the linear regressions which allow the calculation of the TMFs, using the concentration in the chemometers. The grey areas represent the 95% confidence intervals.

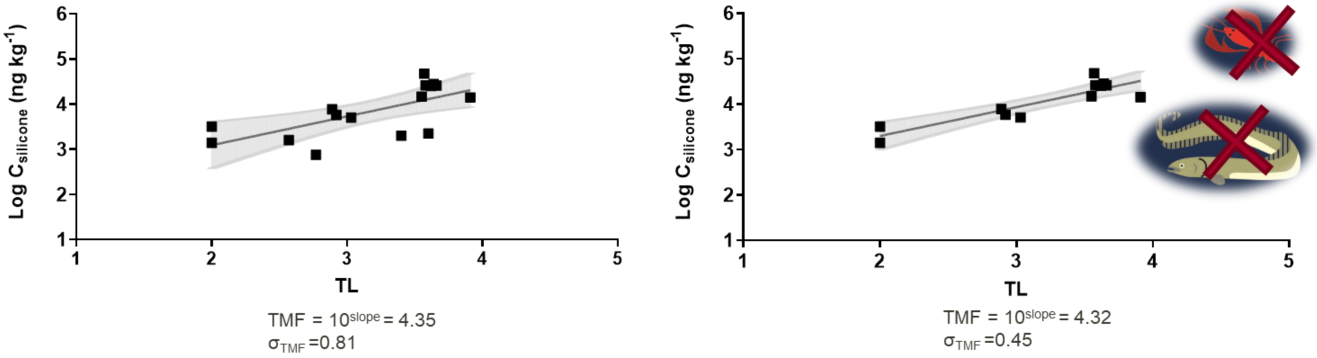

Figure S9. Graphical representation of the linear regressions which allow the calculation of the TMF for PCB153, using the concentration in the chemometers, on the left with eels and crayfish included, and on the right with them excluded. The grey areas represent the 95% confidence intervals and σ_{TMF} is the error propagation of the SD of the slope.

Text S7: Modeled uptake in the chemometer for water sampling with different thicknesses of the water boundary layer (δ).

In order to model the uptake of the studied chemicals into the µm-thin coatings of the chemometers under the scenario of different WBL thicknesses, we used the model proposed by Thompson et al.[9] It is a model for single-phase passive samplers' uptake of HOCs from water and is an analytical solution to Fick's second law applied through an aqueous diffusive boundary layer and a polymer layer. It presents a Fickian approach to model HOCs uptake, originally into polyethylene in sampler strips in aqueous systems, exposed from both sides without flux across the midline of the strip due to symmetry. We have adapted the model to the settings of our study, using the parameters detailed in Table S9 and considering one-side exposure of the silicone in the coated glass jar sampler (the jars are coated on their inner vertical walls, so the coated area presents a cylinder surface), implementing those variations in the original script and applying it using Matlab Version 2024b (MathWorks, USA). Different thicknesses of the WBL (δ) have been modeled as follows: δ = 10 µm as a lower bound for turbulent systems, δ = 50 µm as an agitated laboratory system and δ = 500 µm as upper limit for WBL, considering a quiescent system.[9] Three other intermediate scenarios have been considered, δ = 20 µm, δ = 30 µm and δ = 100 µm. Even if some studies have calculated δ <10µm, we have kept the lower limit of the modeled WBL in δ = 10 µm for turbulent systems.

Figure S10: Mass uptake (M) into the silicone chemometer for water sampling with different thicknesses (δ) of the water boundary layer. The blue line represents the two-phase Fickian model with the best fit value of δ. Model and original script for Matlab, modified from Thompson et al., 2015.[9]

PCB52, thickness of the chemometer =3.5µm

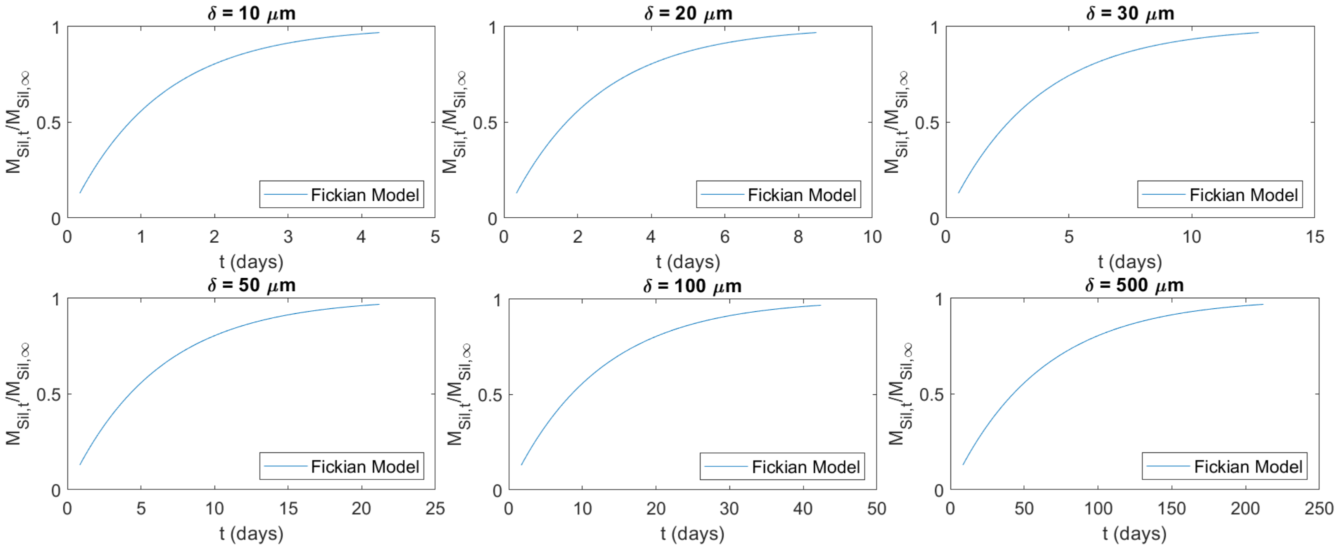

PCB52, thickness of the chemometer =2µm

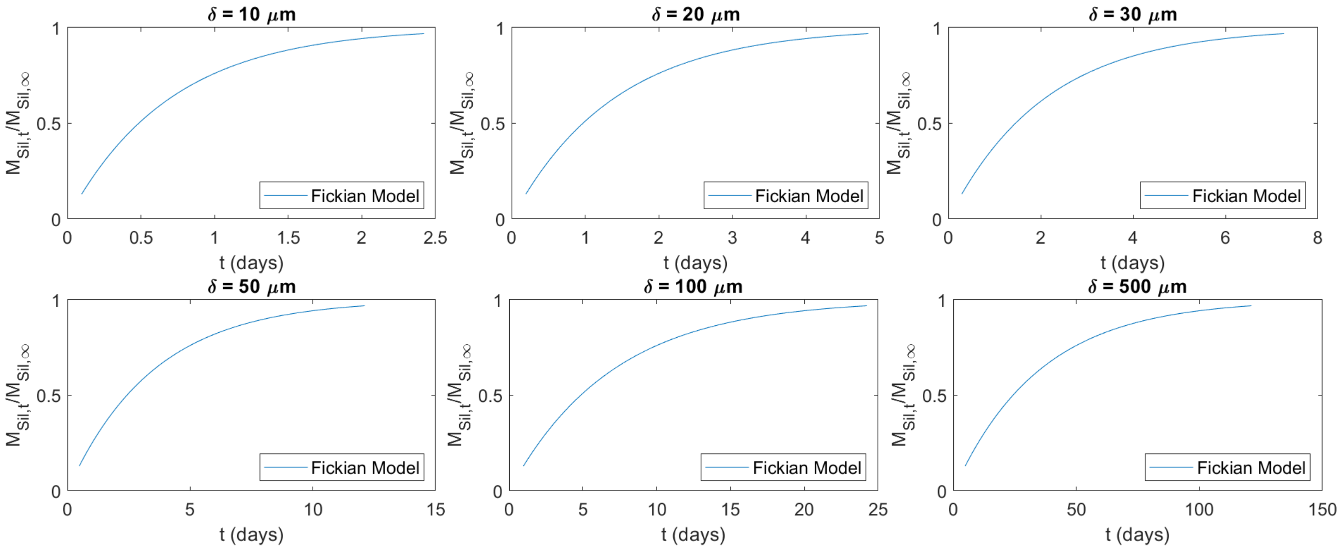

PCB52, thickness of the chemometer =1 μm

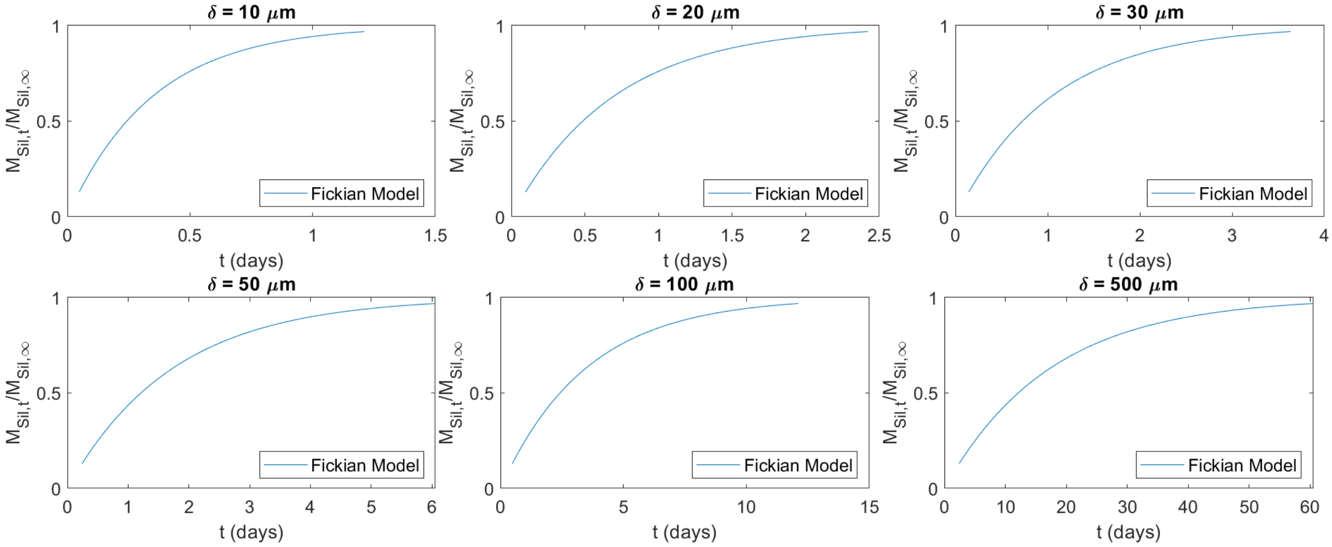

PCB52, thickness of the chemometer =0.6 μm

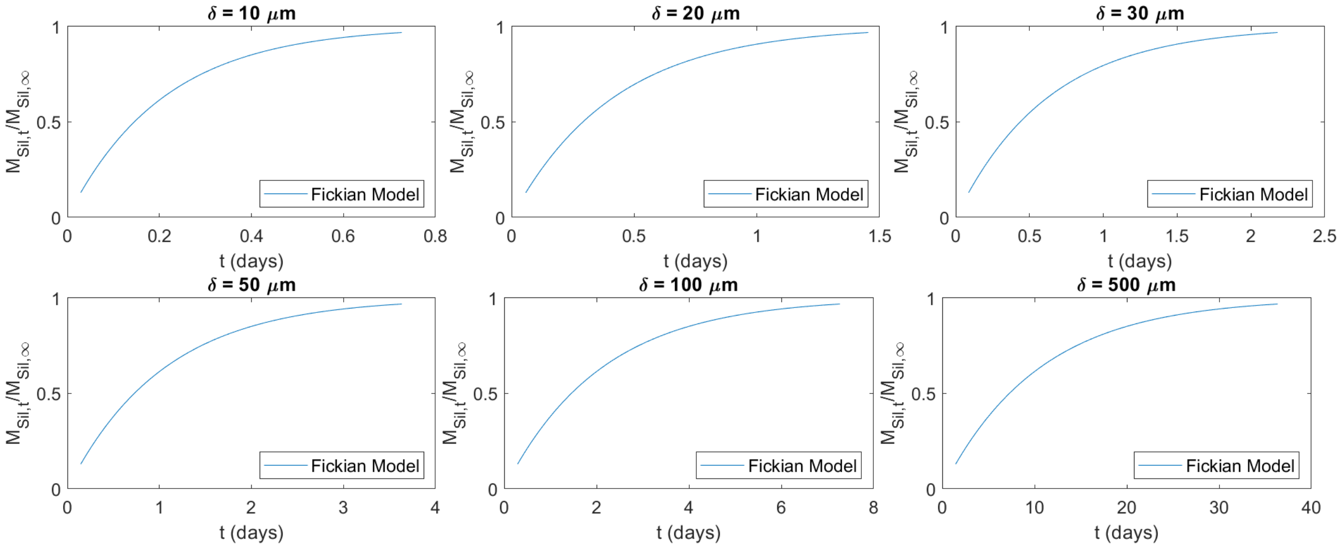

PCB44, thickness of the chemometer =3.5μm

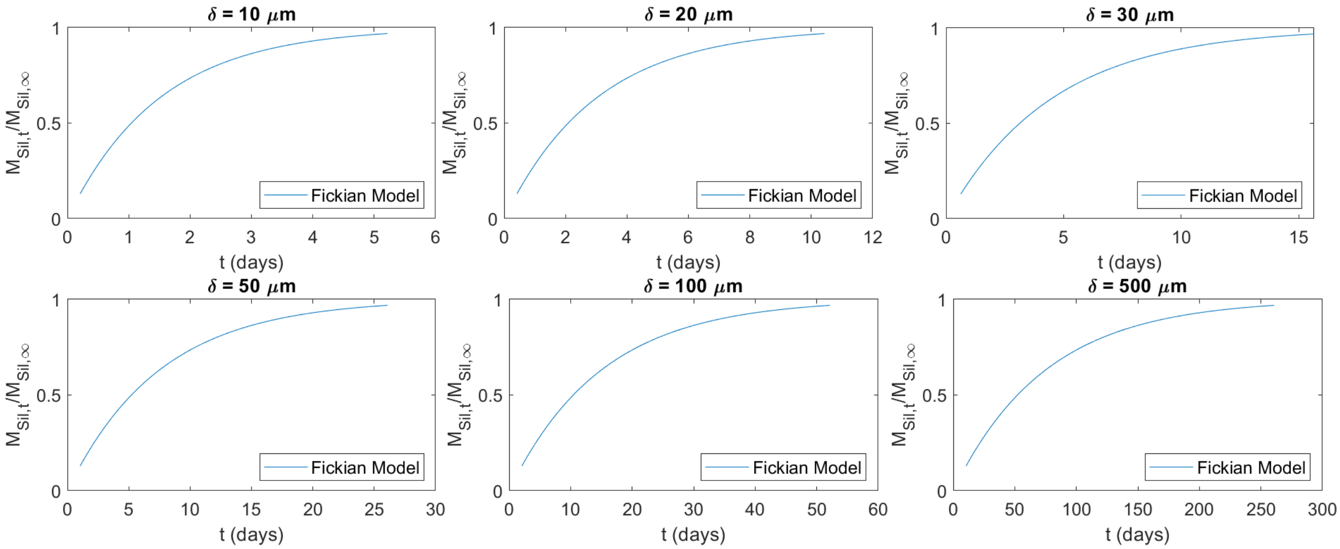

PCB44, thickness of the chemometer =2µm

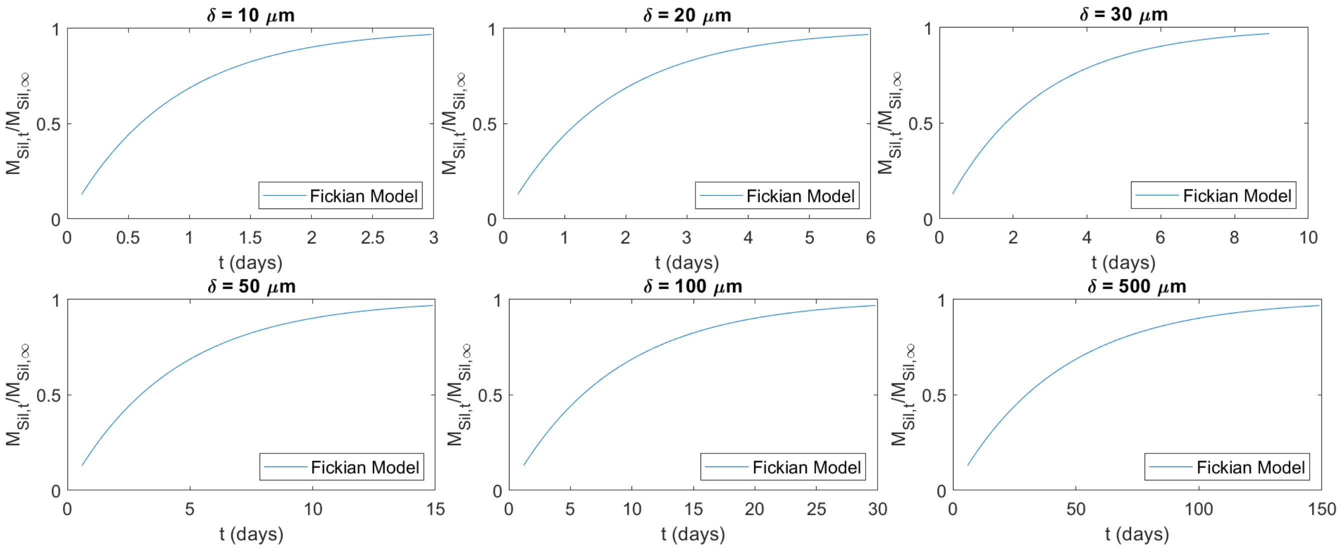

PCB44, thickness of the chemometer =1 µm

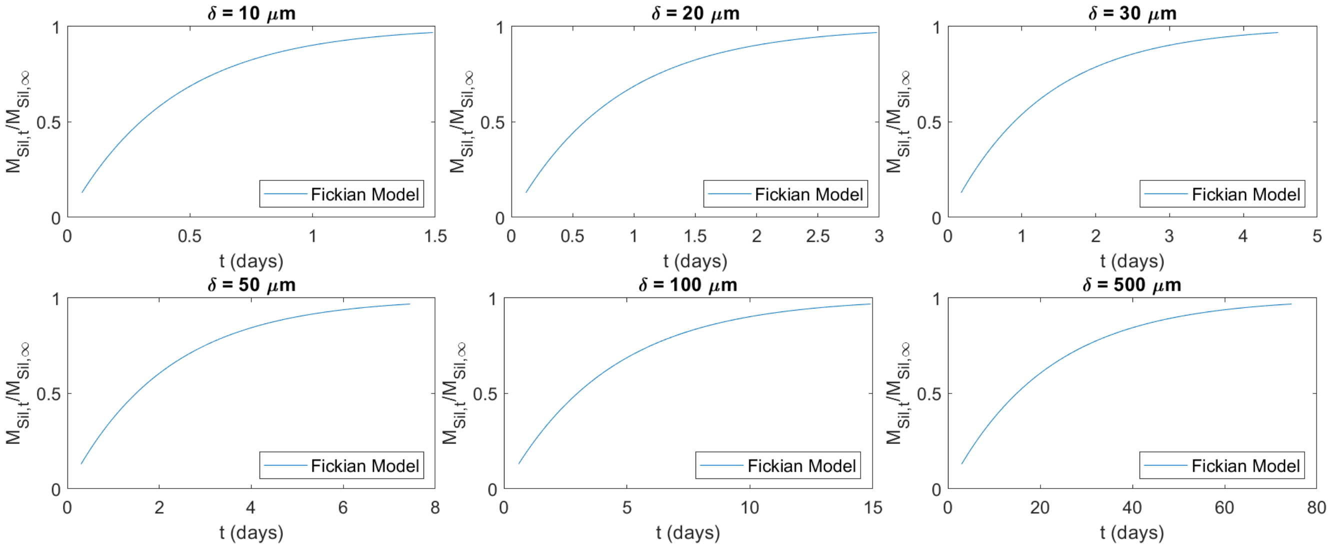

PCB44, thickness of the chemometer =0.6 µm

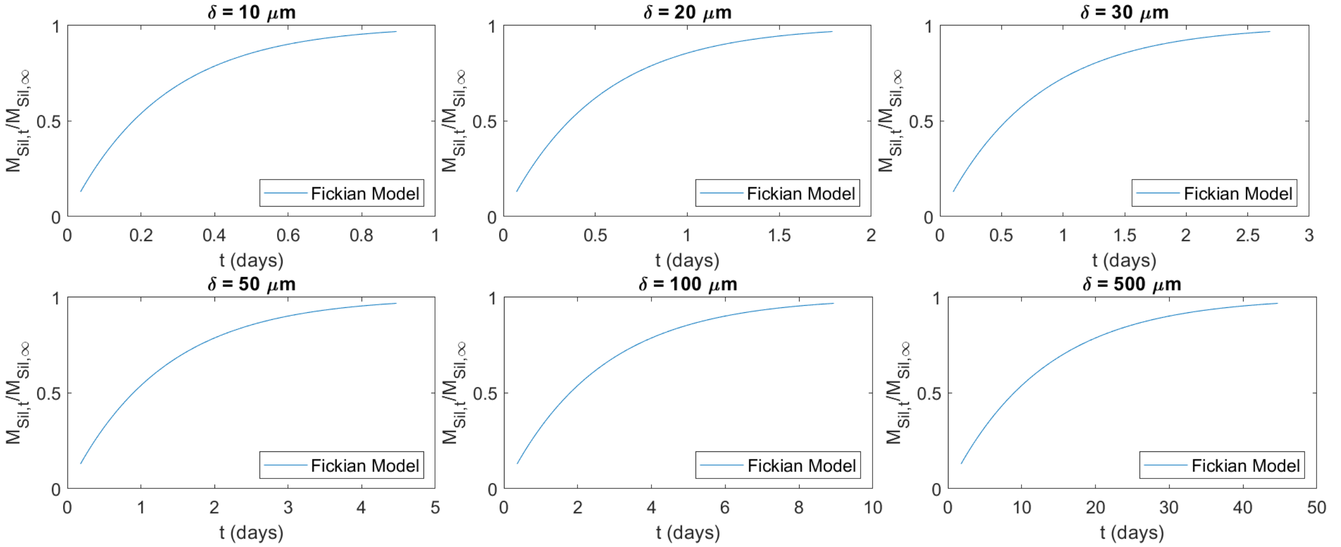

PCB101, thickness of the chemometer =3.5µm

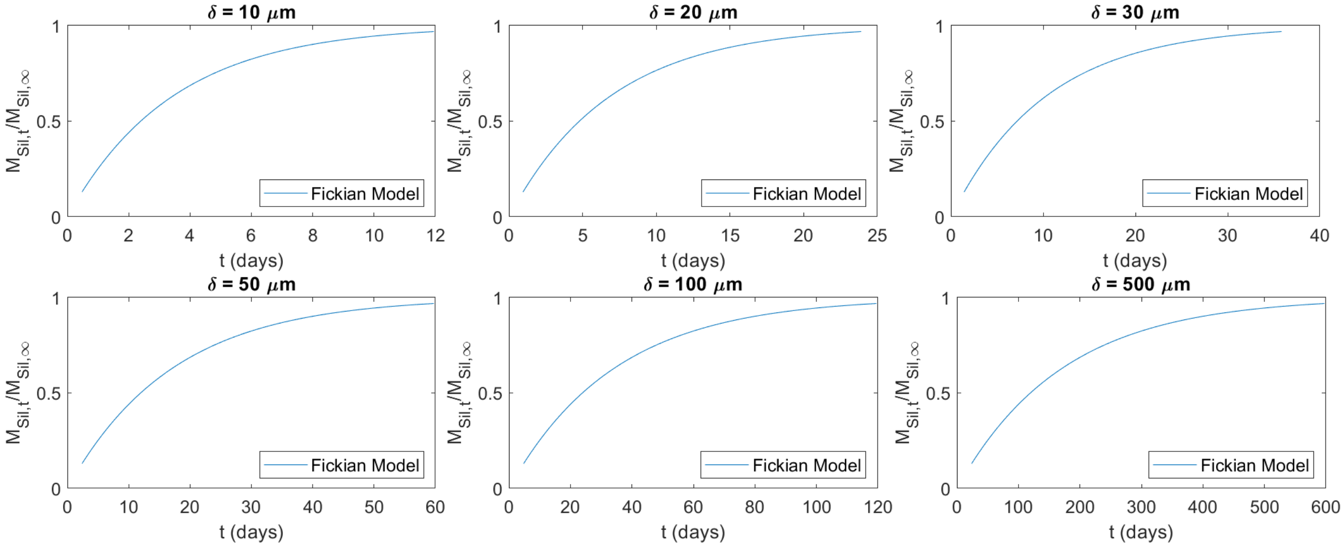

PCB101, thickness of the chemometer =2µm

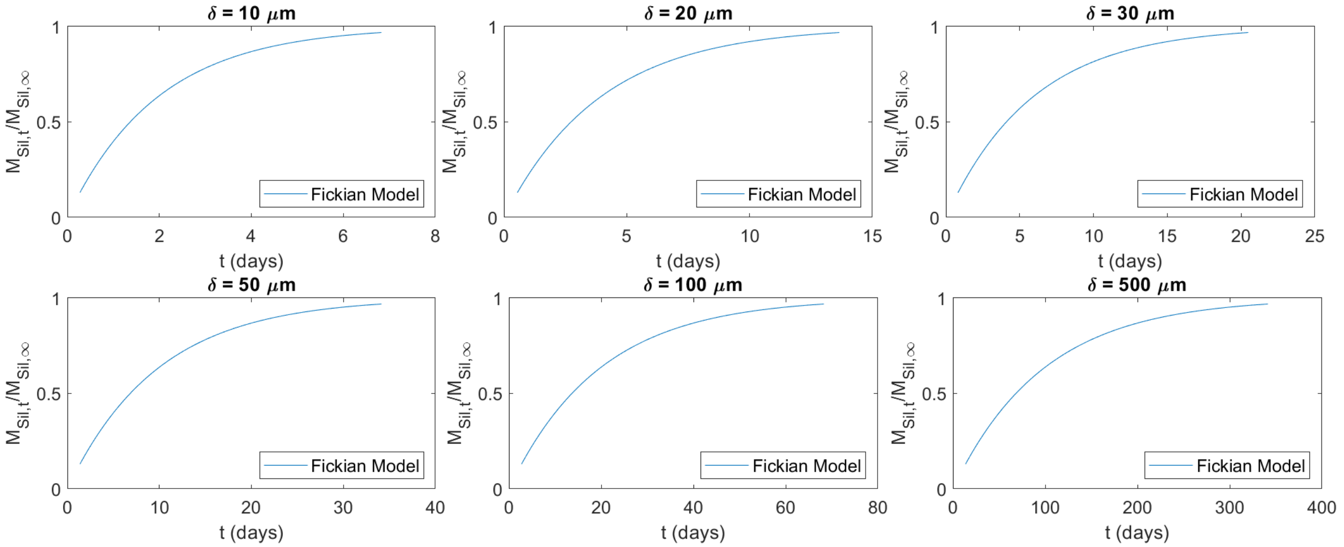

PCB101, thickness of the chemometer =1 µm

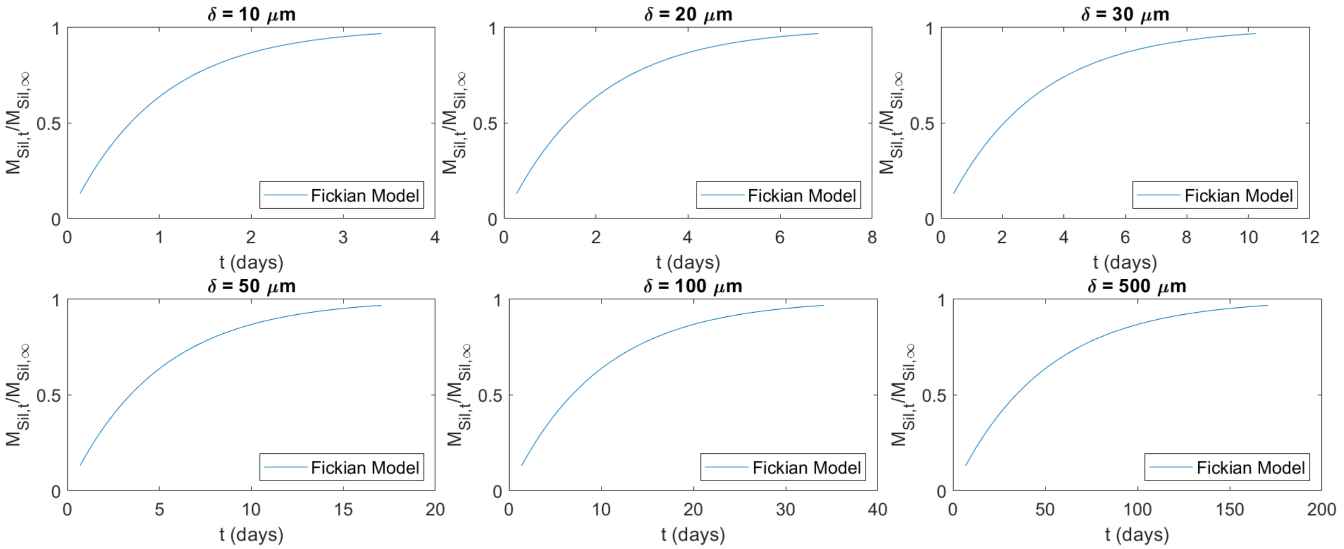

PCB101, thickness of the chemometer =0.6 µm

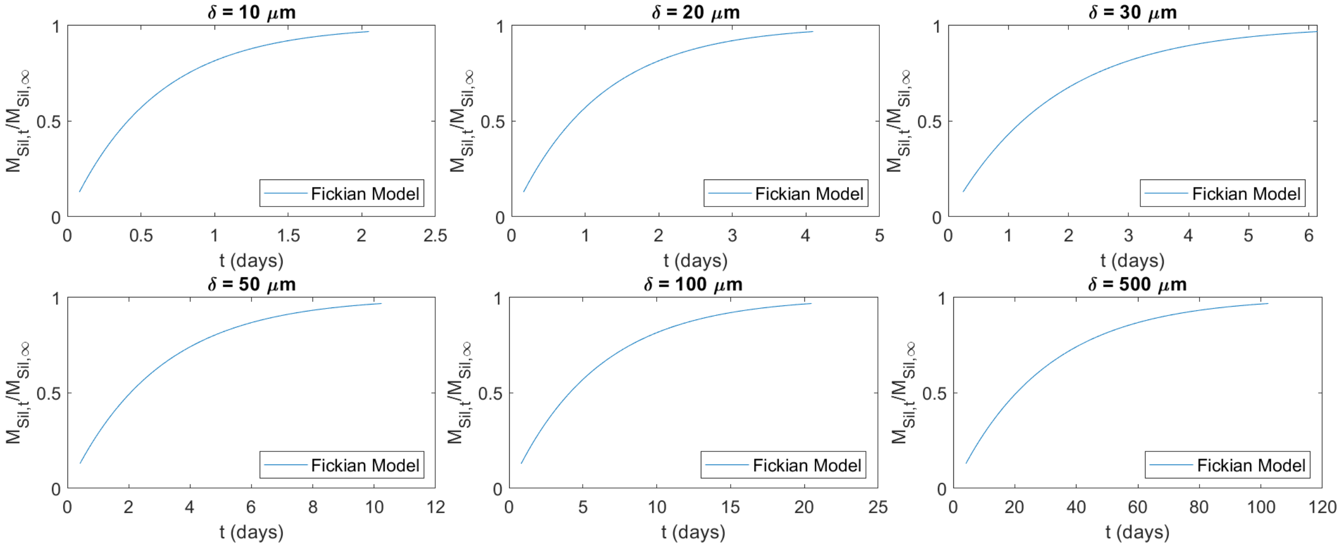

PCB118, thickness of the chemometer =3.5µm

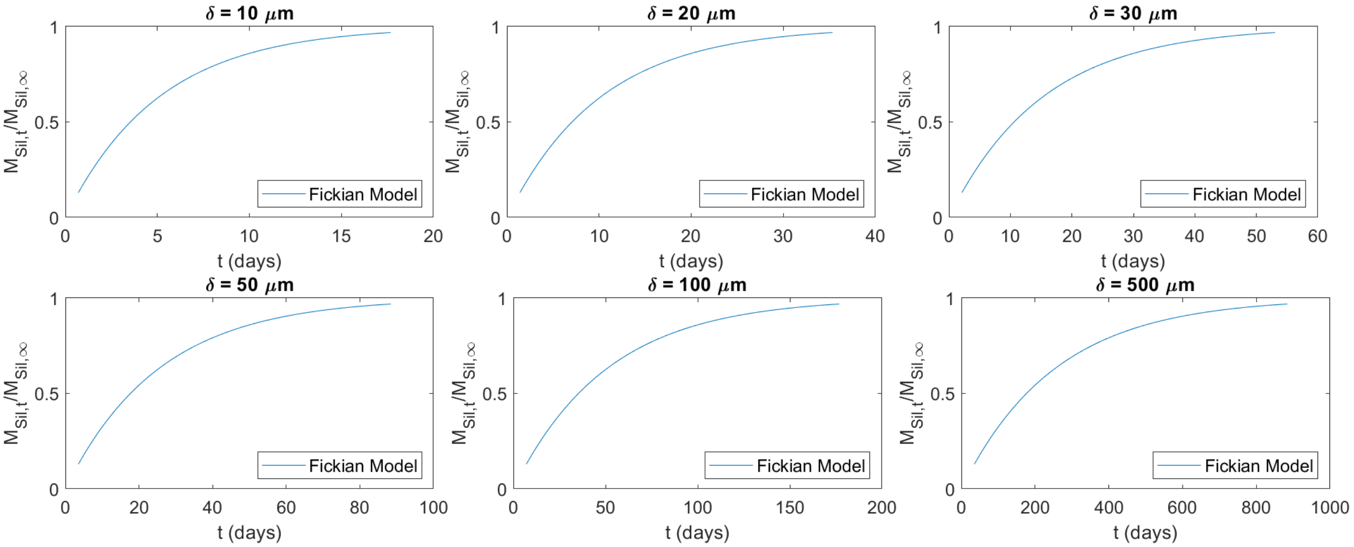

PCB118, thickness of the chemometer =2µm

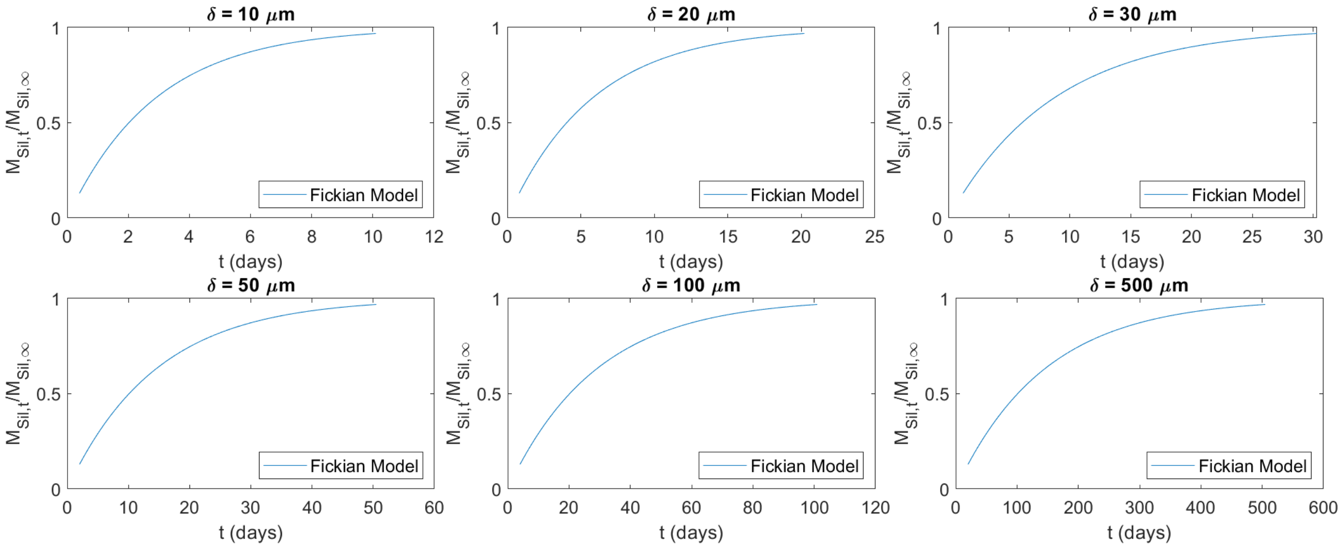

PCB118, thickness of the chemometer =1 µm

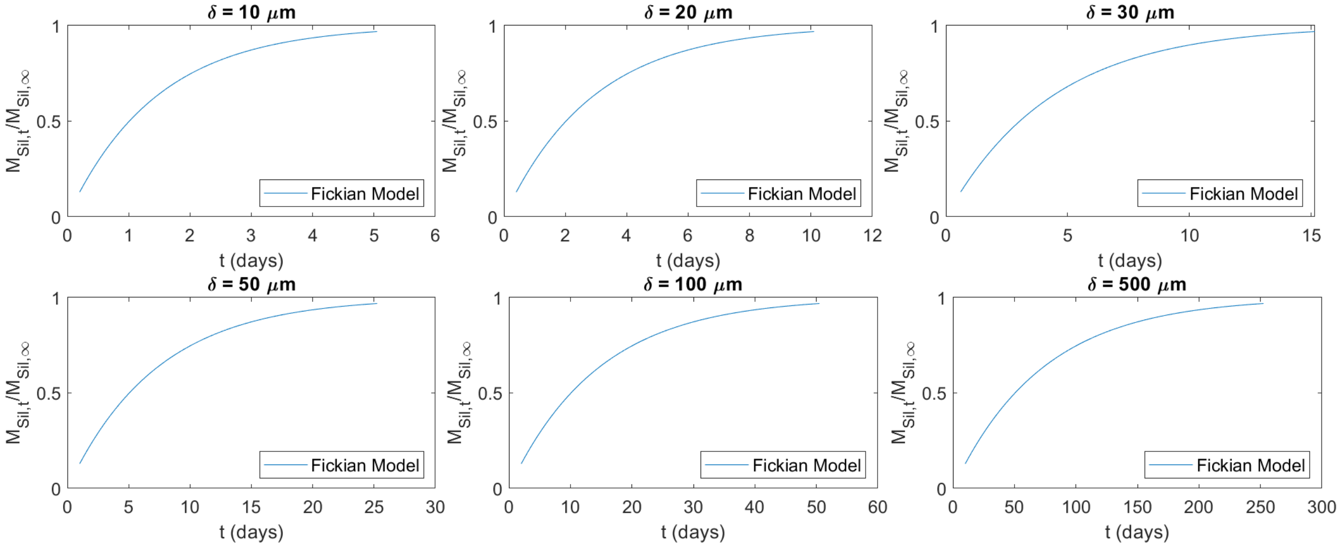

PCB118, thickness of the chemometer =0.6 µm

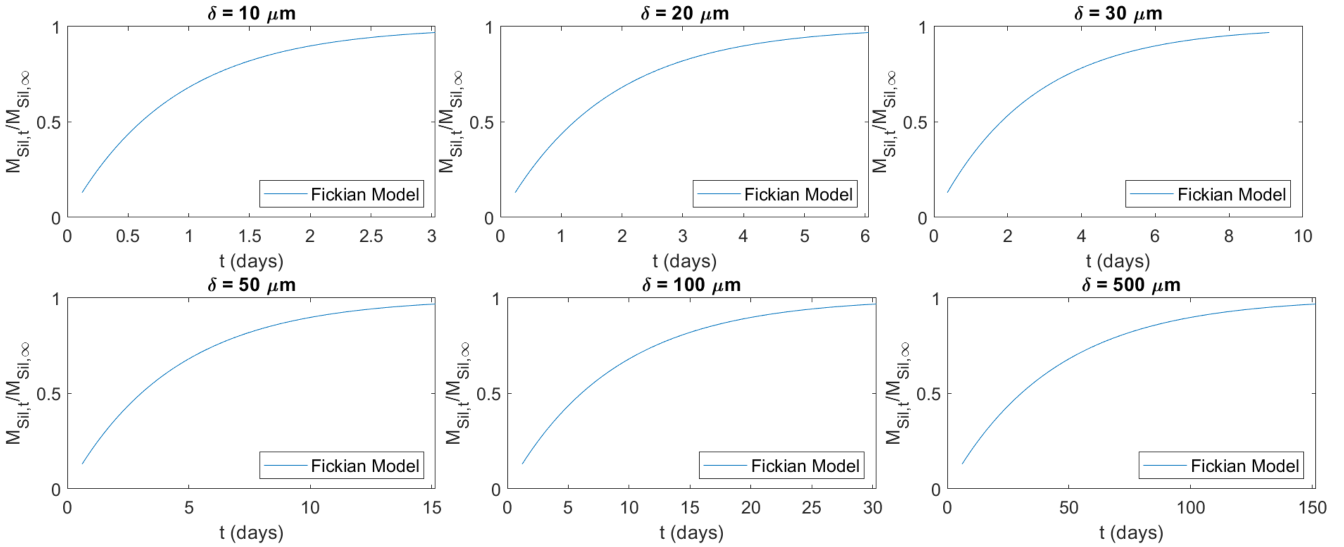

PCB138, thickness of the chemometer =3.5µm

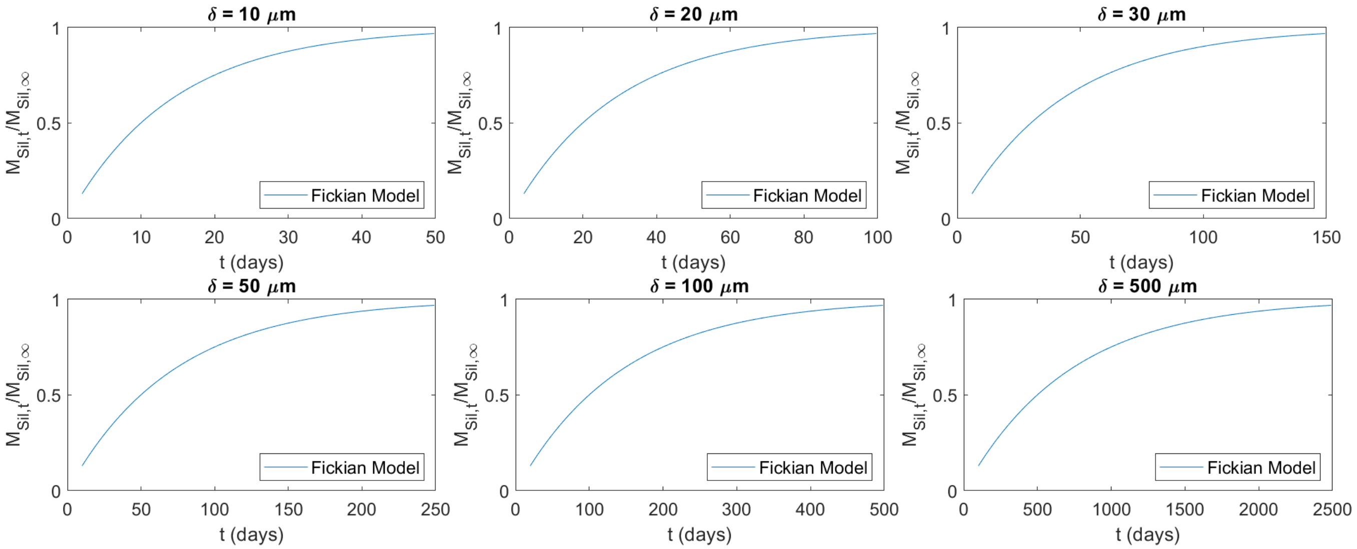

PCB138, thickness of the chemometer =2µm

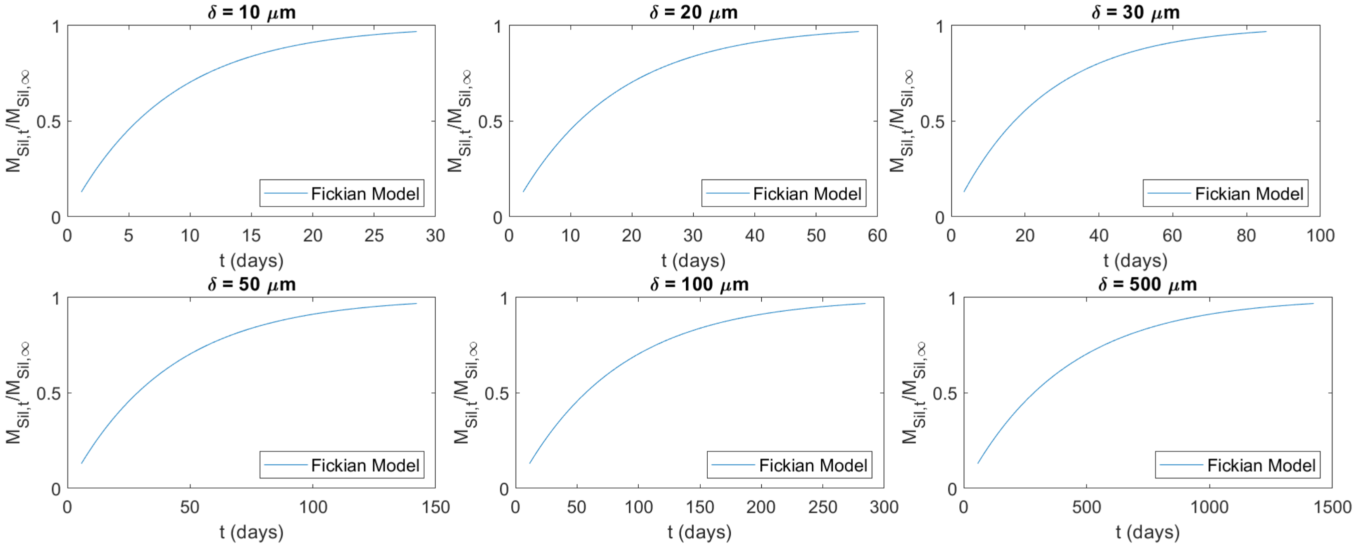

PCB138, thickness of the chemometer =1 µm

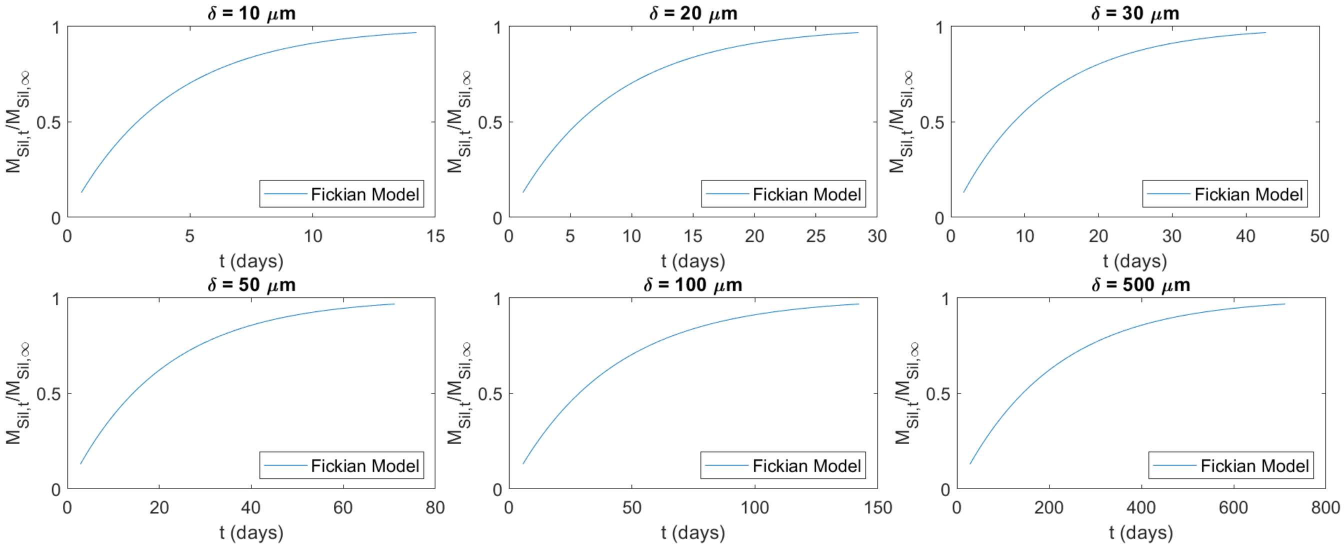

PCB138, thickness of the chemometer =0.6 µm

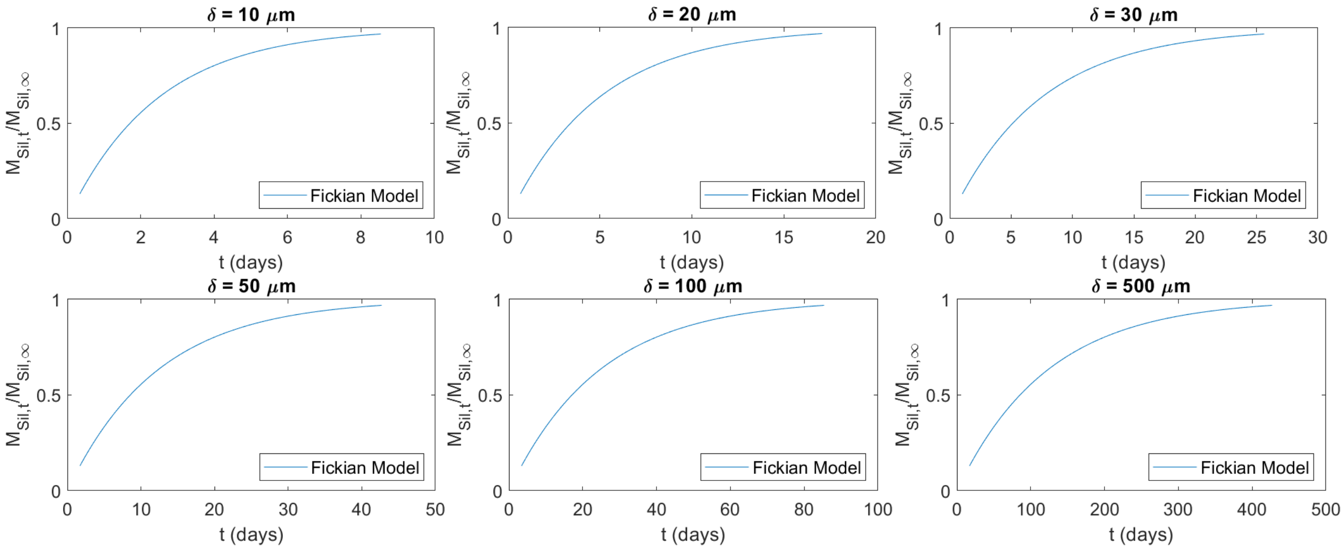

PCB153, thickness of the chemometer =3.5µm

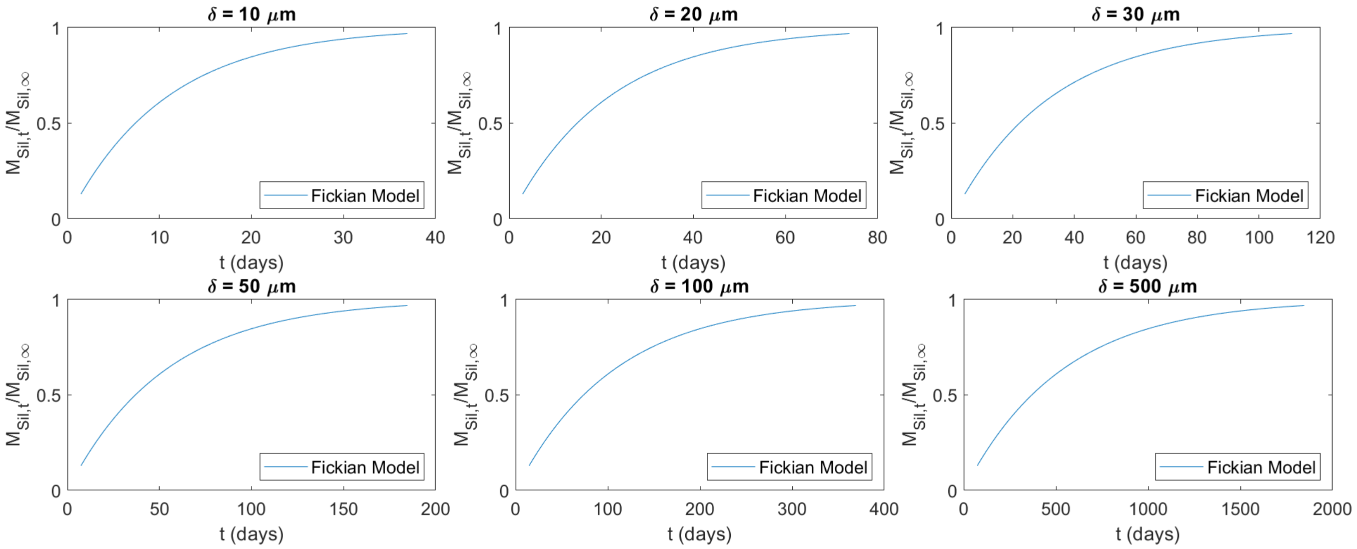

PCB153, thickness of the chemometer =2µm

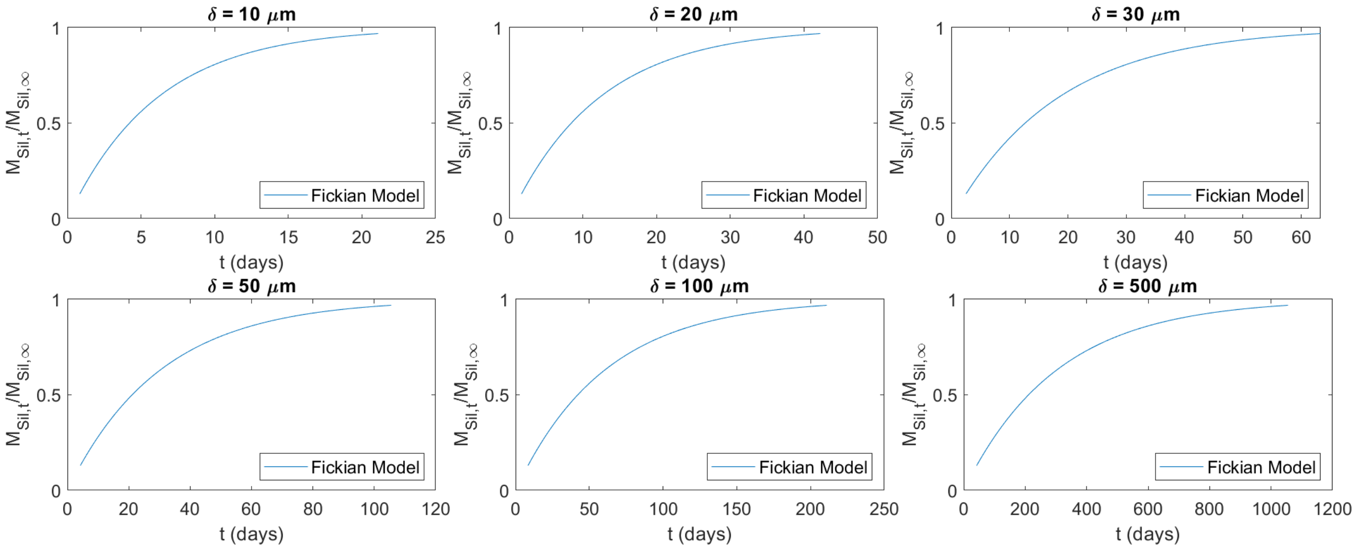

PCB153, thickness of the chemometer =1 µm

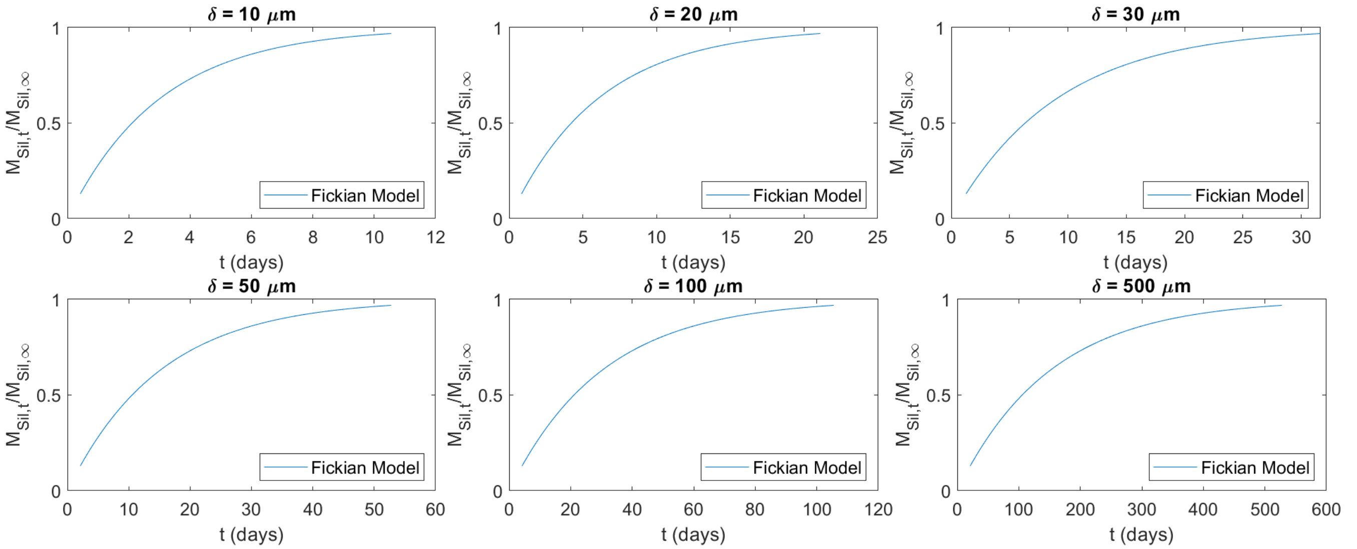

PCB153, thickness of the chemometer =0.6 µm

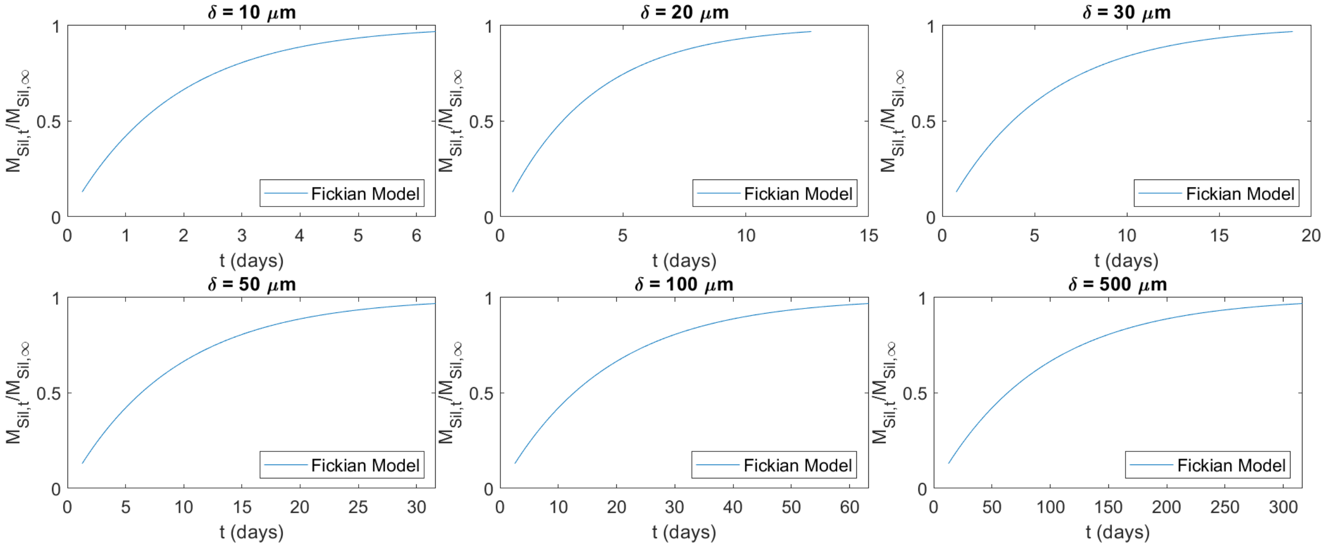

References

(1) Muz, M.; Rojo-Nieto, E.; Jahnke, A. Removing Disturbing Matrix Constituents from Biota Extracts from Total Extraction and Silicone-Based Passive Sampling. *Environ Toxicol Chem* **2021**, *40* (10), 2693–2704. https://doi.org/10.1002/etc.5153.

(2) Reichenberg, F.; Smedes, F.; Jönsson, J. A.; Mayer, P. Determining the Chemical Activity of Hydrophobic Organic Compounds in Soil Using Polymer Coated Vials. *Chem Cent J* **2008**, *2* (1), 1–10. https://doi.org/10.1186/1752-153X-2-8.

(3) Kosfeld, V.; Rüdel, H.; Schlechtriem, C.; Rauert, C.; Koschorreck, J. Food Web on Ice: A Pragmatic Approach to Investigate the Trophic Magnification of Chemicals of Concern. *Environ Sci Eur* **2021**, *33* (1). https://doi.org/10.1186/s12302-021-00530-x.

(4) Jahnke, A.; MacLeod, M.; Wickström, H.; Mayer, P. Equilibrium Sampling to Determine the Thermodynamic Potential for Bioaccumulation of Persistent Organic Pollutants from Sediment. *Environ Sci Technol* **2014**, *48* (19), 11352–11359. https://doi.org/10.1021/es503336w.

(5) Jahnke, A.; Mayer, P.; McLachlan, M. S.; Wickström, H.; Gilbert, D.; MacLeod, M. Silicone Passive Equilibrium Samplers as 'Chemometers' in Eels and Sediments of a Swedish Lake. *Environ Sci Process Impacts* **2014**, *16* (3), 464–472. https://doi.org/10.1039/C3EM00589E.

(6) Walters, D. M.; Jardine, T. D.; Cade, B. S.; Kidd, K. A.; Muir, D. C. G.; Leipzig-Scott, P. Trophic Magnification of Organic Chemicals: A Global Synthesis. *Environ Sci Technol* **2016**, *50* (9), 4650–4658. https://doi.org/10.1021/acs.est.6b00201.

(7) Kidd, K. A.; Burkhard, L. P.; Babut, M.; Borgå, K.; Muir, D. C. G.; Perceval, O.; Ruedel, H.; Woodburn, K.; Embry, M. R. Practical Advice for Selecting or Determining Trophic Magnification Factors for Application under the European Union Water Framework Directive. *Integr Environ Assess Manag* **2019**, *15* (2), 266–277. https://doi.org/10.1002/ieam.4102.

(8) European Union. *CIS-WFD Guidance Document No. 32 On Biota Monitoring (the Implementation of EQSbiota) under the Water Framework Directive Technical Report 2014-083*; 2014. https://doi.org/10.2779/833200.

(9) Thompson, J. M.; Hsieh, C. H.; Luthy, R. G. Modeling Uptake of Hydrophobic Organic Contaminants into Polyethylene Passive Samplers. *Environ Sci Technol* **2015**, *49* (4), 2270–2277. https://doi.org/10.1021/es504442s.
